# *Cochlospermum angolense* Welw ex Oliv: Phytochemical Profile, Antioxidant Activity, and Therapeutic Prospects

**DOI:** 10.3390/molecules30132768

**Published:** 2025-06-27

**Authors:** Nsevolo Samba, Abdy Morales Barrios, Estela Guerrero De León, Cesar Raposo, Radhia Aitfella Lahlou, Joana Curto, Jesus M. Rodilla, Alejandro M. Roncero, David Diez, Lúcia Silva

**Affiliations:** 1 Chemistry Department, University of Beira Interior, 6201-001 Covilhã, Portugal; nsevolo.samba@ubi.pt (N.S.); ra.aitfella@ubi.pt (R.A.L.); jmrc@ubi.pt (J.C.);; 2 Department of Clinical Analysis and Public Health, University Kimpa Vita, Uige 77, Angola; 3 Fiber Materials and Environmental Technologies (FibEnTech-UBI), University of Beira Interior, 6201-001 Covilhã, Portugal; 4 Departamento de Farmacologías, Facultad de Medicina, Universidad de Panamá, Panama City 7096, Panama; moba245@gmail.com (A.M.B.); guerrerodleon@gmail.com (E.G.D.L.); 5 Centro de Investigaciones Psicofarmacológicas, Universidad de Panamá, Panama City 7096, Panama; 6 Mass Spectrometry Service, NUCLEUS, University of Salamanca, 37005 Salamanca, Spain; raposo@usal.es; 7 RISE-Health, Faculdade de Ciências da Saúde, Universidade da Beira Interior, Av. Infante D. Henrique, 6200-506 Covilhã, Portugal; 8 Departamento de Química Orgánica, Facultad de Ciencias Químicas, Universidad de Salamanca, Avda de los Caídos s/n, 37008 Salamanca, Spain; alexmaron@usal.es

**Keywords:** *Cochlospermum angolense*, roots, leaves, bark, antioxidant activity, HPLC-ESI-MSn

## Abstract

The phytochemical investigation and evaluation of the antioxidant activity of the leaves, bark, and roots of *Cochlospermum angolense* Welw ex Oliv—a valued plant that is widely used in traditional Angolan medicine—hold significant importance. Compounds were extracted from the aforementioned plant using acetone and ethanol and identified by HPLC-ESI-MSn. Both extracts demonstrated notable abilities to scavenge 2,2-diphenyl-1-picrylhydrazyl, nitric oxide, and superoxide radicals, as well as to inhibit lipid peroxidation. A HPLC analysis revealed a diverse array of bioactive compounds, including flavonoids, phenols, alkaloids, quinones, and terpenes, which help neutralize free radicals and protect cells against oxidative stress, thereby contributing to the prevention of various diseases. Moreover, the acetone and ethanol extracts proved to be excellent sources of antioxidants. For the first time, the present study identified new compounds never reported in this species, such as (+)-abscisic acid, angustine B, pinobanksin, dihydrogenistein, (−)-8-prenylnaringenin, isoquercetin, samandarine, dihydromyricetin, and eupatoriocromene, in the leaves, bark, and roots, marking a significant advance in the chemical characterization of *C. angolense*. These findings enhance our understanding of the bioactive phytochemicals and antioxidant properties of *C. angolense* and open new avenues for future therapeutic and pharmacological research, further supporting its traditional use in Angolan medicine.

## 1. Introduction

Biodiversity is fundamental to human livelihoods and overall well-being [1,2]. Throughout history, people have maintained a close relationship with their natural environment, relying on its diverse flora for food, shelter, and medicine [3,4]. The enduring allure of nature lies in its potential to yield chemotherapeutic agents, with plant-derived natural compounds continuing to serve as vital sources of new leads in drug discovery [5,6,7]. Plants have consistently met essential human needs, not only by providing shelter, clothing, food, flavors, and fragrances, but also forming the basis of traditional medicine, from which many modern drugs have emerged [8]. Consequently, pharmacological and phytochemical research is critical for both uncovering new therapeutic agents and preserving medicinal plants as part of our cultural and scientific heritage. Medicinal plants rich in bioactive compounds are highly valued for their ability to reduce the risk of chronic diseases and promote overall health [9].

Angola is home to a rich diversity of medicinal plants whose full potential remains largely untapped [10]. The country’s varied flora represents a significant opportunity for discovering new secondary metabolites with therapeutic value, particularly given the longstanding traditional use of these plants. Known for their antioxidant properties, many of these species can help reduce disease incidence by neutralizing free radicals, thereby protecting cells and potentially preventing oxidative stress-related disorders [2,11]. The importance of these medicinal plants lies in their chemical constituents, which exhibit a range of biological activities and are broadly classified into primary and secondary metabolites [12,13]. These metabolites have garnered considerable attention for their potential as pharmaceutical agents, food additives, and valuable chemicals for human use. The bioactive compounds extracted from medicinal plants are rich in antioxidants, which are beneficial for developing treatments for various diseases [14,15]. Antioxidants prevent the oxidation process, thereby inhibiting the formation of free radicals that can damage healthy cells and contribute to chronic conditions such as cardiovascular diseases, diabetes, and cancer. In addition, these compounds play a crucial role in wound healing by promoting the repair of damaged skin. Several studies have suggested that a significant portion of the therapeutic value of plant extracts can be attributed to their antioxidant activity [16].

Members of the *Cochlospermum* genus are renowned for their richness in secondary metabolites, notably phenolic compounds, flavonoids, lignans, carotenoids, and sterols, which underpin their extensive medicinal applications [17,18]. The phytochemicals found in these plants, including those in the leaves, bark, and roots of *Cochlospermum angolense* Welw ex Oliv, exhibit potent antioxidant properties—an essential feature, given that many diseases are associated with free radicals produced during normal physiological processes and counteracted by plant-derived antioxidants [19,20]. Phenolic compounds in particular have been extensively studied for their health benefits. Characterized by multiple hydroxyl groups on aromatic rings, these secondary metabolites contribute to antioxidant, antimicrobial, and anti-inflammatory activities [21]. Despite the high antioxidant content typical of these species, few studies have directly compared the pharmacological and phytochemical properties of acetone and ethanol extracts from the roots, leaves, and bark of *C. angolense*. This Angolan plant is regarded as phytotherapeutic and is widely used by rural populations to both cure and prevent various ailments. For instance, beverages or infusions made from the roots and bark are traditionally used to combat intestinal diseases, parasitic infections, malaria, diabetes, and hepatitis, while the leaves are employed in baths to treat fevers, especially in children [20,22,23].

This research offers valuable insights into the traditional use of plants by local communities, highlighting their potential for both medicinal applications and biodiversity conservation. The study aimed to evaluate the antioxidant capacity of acetone and ethanol extracts obtained from the leaves, bark, and roots of *C. angolense*. Antioxidants are vital because they neutralize free radicals, which, if left unchecked, can cause cellular damage and contribute to the development of chronic diseases.

To thoroughly assess the antioxidant potential of *C. angolense*, our research utilized two complementary approaches. First, the antioxidant activity of the extracts was determined through a series of assays that measured the ability of the compounds to scavenge harmful free radicals. Second, the phytochemical composition of the extracts was meticulously analyzed using High-Performance Liquid Chromatography coupled with Electrospray Ionization and Tandem Mass Spectrometry (HPLC-ESI-MSn). This advanced analytical technique enabled the precise identification and characterization of a broad range of bioactive compounds, including flavonoids, phenolic acids, and other secondary metabolites known for their health-promoting properties. Detailed phytochemical profiling not only confirmed the presence of compounds associated with potent antioxidant activity but also provided a scientific basis for the traditional medicinal use of *C. angolense*. By documenting the specific bioactive constituents, the study paves the way for further pharmacological research and the potential development of novel therapeutic agents. Moreover, these findings underscore the importance of preserving local biodiversity and traditional knowledge, which are invaluable resources for sustainable healthcare innovations and conservation efforts.

## 2. Results and Discussion

For this research project, High-Performance Liquid Chromatography coupled with Electrospray Ionization and Tandem Mass Spectrometry (HPLC-ESI-MSn) was employed to comprehensively identify the phytochemical compounds present in the acetone and ethanol extracts obtained from the leaves, bark, and roots of the medicinal plant *C. angolense*. The primary goal was to correlate these compounds with their potential antioxidant properties [24]. The extraction process was carried out using standardized protocols to ensure reproducibility and a high yield of metabolites. Phytochemicals were identified based on their molecular formulas, retention times, and fragmentation patterns and were subsequently classified into distinct organic classes. The distribution of these compounds across the different plant parts was meticulously recorded in Supplementary File, allowing for a detailed comparative analysis and providing insights into structure–activity relationships.

The analysis resulted in the successful identification of 520 phytochemicals across the samples. Among these, 61 bioactive compounds were specifically associated with the antioxidant activity of *C. angolense* (Supplementary File). These key compounds primarily belong to the classes of flavonoids, phenolics, alkaloids, terpenoids, quinones, and tannins. Detailed spectral analysis and observed fragmentation patterns provided insights into the structural features of these molecules, which are known to scavenge free radicals and inhibit lipid peroxidation. Numerous studies have reported that these classes of compounds exhibit a range of biological activities—including antioxidant, antimicrobial, anti-inflammatory, antiplasmodial, and anticancer effects—in various medicinal plants [18,20,25,26]. For example, the alkaloids identified in this study have been shown to exert antiplasmodial activity by inhibiting protein synthesis in *Plasmodium falciparum* [27]. In addition to these major groups, comprehensive phytochemical profiling also revealed secondary metabolites such as aldehydes, coumarins, peptides, and esters, which may act synergistically to enhance the overall antioxidant effect. The precise quantification and structural elucidation achieved with HPLC-ESI-MSn underscore the robustness and reliability of this analytical method. Overall, the wide spectrum of antioxidant phytochemicals identified in *C. angolense* highlights their critical role in neutralizing free radicals and mitigating oxidative stress [11,28]. The discovery of the phytochemicals listed in Table 1 provides a solid scientific basis for the traditional medicinal use of *C. angolense* and substantiates the need for further pharmacological research, as well as the development of new therapeutic agents (Table 1).

After a detailed analysis of the bioactive phytochemicals present in the leaves, bark, and roots of the medicinal plant *C. angolense*, a diverse range of compounds with potential therapeutic and pharmacological applications was identified. These compounds, comprehensively listed in Supplementary File, belong to several organic classes, including flavonoids, phenols, alkaloids, sesquiterpenoids, quinones, and aldehydes—each known for its unique bioactive properties.

**Figure 1:**
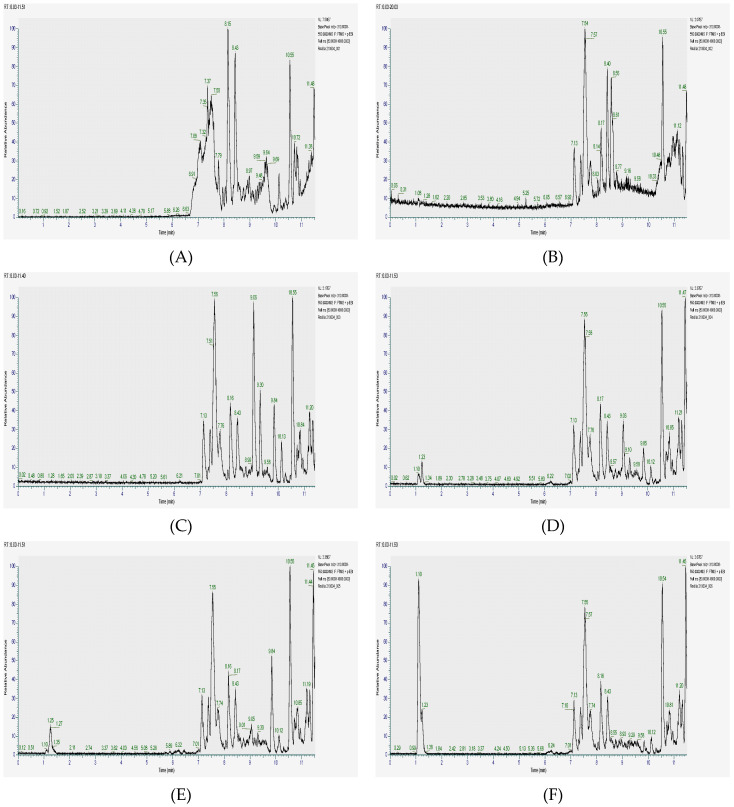
HPLC-ESI-MSn Chromatograms of *Cochlospermum angolense* extracts. (**A**) Acetone extract of the leaves; (**B**) Ethanolic extract of leaves; (**C**) Acetone extract of barks; (**D**) Ethanolic extract of barks; (**E**) Acetone extract of roots; (**F**) Ethanolic extract of roots.

The extraction was performed using both acetone and ethanol, and the resulting extracts were analyzed using HPLC-ESI-MSn. The chromatograms obtained (Figure 1) not only provided a visual representation of complex phytochemical profiles but also enabled the precise identification of individual compounds based on their molecular formulas, retention times, and fragmentation patterns. The data revealed that the highest concentrations of flavonoids and phenols were consistently found across all three plant parts, suggesting that these metabolites play a significant role in the overall antioxidant and therapeutic potential of *C. angolense*.

A closer comparison of the different plant parts indicated that the leaves are particularly enriched in flavonoids and phenolic compounds, which are well known for their free radical scavenging and anti-inflammatory activities. In contrast, bark exhibited a broader diversity of alkaloids and quinones, compounds that have been associated with antimicrobial and anticancer effects. The roots, on the other hand, displayed a unique chemical profile characterized by a significant presence of sesquiterpenoids combined with phenols, hinting at specialized roles in traditional remedies and potential synergistic effects in pharmacological applications. These findings not only align with previous studies on related species within the same family [29,30] but also underscore the importance of exploring each plant part separately to fully understand its distinct phytochemical contributions. The detailed profiling achieved in this study provided a consistent result for the traditional medicinal use of *C. angolense* and opens new avenues for further research into its potential as a source of novel therapeutic agents.

All extracts of *Cochlospermum angolense* Welw ex Oliv exhibited a remarkable diversity of phytochemical compounds endowed with potent antioxidant activities, as demonstrated by multiple in vitro assays [29,31]. The extracts contained high concentrations of bioactive compounds, notably flavonoids, phenols, terpenes, and alkaloids. In-depth analysis revealed that the flavonoid fraction was particularly abundant, with specific compounds such as quercitrin, astragalin, vitexin, apigetrin, quercetin-3′-glucuronide, isoquercetin, morin, quercetin-3-arabinoside, kaempferol, (+)-dihydrokaempferol, naringenin, pinobanksin, angustine B, licochalcone, morachalcone, and (−)-8-prenylnaringenin being identified [21,32,33]. These flavonoids not only exhibit significant elimination of free radical abilities but also modulate cellular signaling pathways, contributing to anti-inflammatory, antimicrobial, and anticancer effects by interfering with key mechanisms involved in cell proliferation and apoptosis [9,34]. In addition to flavonoids, the phenolic compounds present in the extracts are known for their efficiency in donating hydrogen atoms or electrons to neutralize free radicals, thereby stabilizing radical intermediates and preventing oxidative stress [18,35]. Terpenes and alkaloids further enrich the bioactive profile, offering complementary anti-inflammatory and antimicrobial properties. The synergistic interactions among these diverse classes of compounds likely amplify the overall antioxidant potential of *C. angolense*, enhancing its efficacy in protecting cells from oxidative damage. Recent studies have also suggested that these phytochemicals can influence gene expression related to oxidative stress responses and apoptotic pathways, thereby playing a role in the prevention of chronic diseases such as cardiovascular disorders, neurodegenerative conditions, and cancer [18,20,36,37].

Among the phenolic phytochemicals identified in *C. angolense*, several compounds exhibit notable antioxidant activity [38,39]. These include 4-heptylresorcinol, antiarol, embelin, zingerone, 5-heptylresorcinol, 3-O-methylellagic acid, oleuropeinol (also known as oleuropein), and (+)-[6]-gingerol. These phenolic compounds are known for their ability to neutralize reactive oxygen species (ROS), thereby mitigating oxidative stress at the cellular level and contributing to the prevention of chronic and degenerative diseases.

In addition to phenolics, several alkaloids identified in this study—including samandarine, evocarpine, hygrine, and elaeocanine C—have demonstrated antioxidant potential along with other biological activities, such as antimicrobial, anti-inflammatory, and antiplasmodial effects [40]. These findings are in line with previous research on the leaves and stem bark of *C. gossypium*, which also confirmed the presence of bioactive alkaloid phytochemicals with a wide range of pharmacological properties [40]. Moreover, the acetone and ethanol extracts of *C. angolense* revealed the presence of carboxylic acids, such as 4-pyrimidinecarboxylic acid, 3-hydroxysebacic acid, and 3,5-dihydroxydecanoic acid, as well as terpenoids like farnesoic acid and valerenic acid. These compounds are well-documented for their antioxidant effects, particularly their ability to interrupt oxidative chain reactions by stabilizing radical intermediates and protecting lipids, proteins, and nucleic acids from oxidative damage [41].

Notably, the highest concentrations of flavonoids and phenols were consistently found in the leaves, bark, and roots of *C. angolense*, reinforcing the idea that all parts of the plant contribute significantly to its antioxidant capacity. These results are consistent with findings from studies on *C. religiosum*, a closely related species, which also demonstrated high levels of phenolic and flavonoid compounds in multiple plant organs [42]. Altogether, these findings validate the traditional use of *C. angolense* and provide a strong scientific foundation for its potential therapeutic applications in managing oxidative stress-related conditions.

In this study, the antioxidant capacity of *C. angolense* samples was evaluated using several assays, including DPPH^•^, nitric oxide (NO^•^), superoxide (^•^O_2_^−^), and lipid peroxidation assays, with the detailed results presented in Table 2. The tested extracts, comprising an acetone extract of leaves (L.E.A), ethanolic extract of leaves (L.E.E), acetone extract of bark (B.E.A), ethanolic extract of bark (B.E.E), acetone extract of roots (R.E.A), and ethanolic extract of roots (R.E.E), revealed significant differences in terms of antioxidant activity. The extraction solvent notably influenced both the phytochemical profile and the antioxidant properties of the extracts, as demonstrated by the differences observed in Table 2.

Our findings indicated that significant antiradical components are present in the leaves, bark, and roots of *C. angolense*, which contribute to neutralizing free radicals and protecting cells from oxidative damage, thereby playing a potential role in the prevention of various diseases (Table 2). In this investigation, we employed multiple assays to comprehensively evaluate the antioxidant capacity of the extracts. Extensive literature has described the mechanisms of action and the relationships between phytochemical activity—including that of flavonoids, phenols, tannins, alkaloids, and terpenes—and antioxidant potential [34,43,44]. Antioxidant activity was categorized as high (>70% inhibition), intermediate (40–70% inhibition), or low (<40% inhibition.

The results in Table 2 are expressed in terms of maximum inhibition (Emax, %) and IC_50_ (mg/mL) values for each assay. Certain IC_50_ values were not automatically determined by the GraphPad Prism 10 (version 10.4.2) due to limitations in dose–response curve fitting. In cases where data were not determined (nd), the reason was that the concentration range tested did not reach 50% inhibition of the effect, indicating that the extract did not show effective antiradical activity within the range evaluated.

Notably, the leaf extracts (from both ethanol and acetone extractions) exhibited high Emax values in the DPPH assay, comparable to those of reference antioxidants such as quercetin and curcumin, indicating strong free radical scavenging activity. Although the bark and root extracts also demonstrated significant antioxidant activity, their Emax values were slightly lower than those of the leaf extracts. In the nitric oxide assay, the ethanolic leaf extract showed considerably higher antioxidant activity (Emax of 63.3%) compared to the acetone extract (Emax of 34.8%), while the bark and root extracts displayed moderate activity (Table 2). In the superoxide (O_2_^•−^) assay, the bark extracts from both solvents exhibited particularly high Emax values, indicating an excellent capacity to neutralize superoxide radicals, although the leaf and root extracts were somewhat less effective in this assay (Table 2).

Finally, in the lipid peroxidation assay, all extracts demonstrated significant antioxidant activity, with the leaf extracts performing exceptionally well and showing Emax values close to those of the reference antioxidants. Overall, these results confirm that the leaves, bark, and roots of *C. angolense* are rich sources of antioxidants that can counteract oxidative stress and may contribute to the prevention of oxidative stress-related diseases.

The results indicate that both the acetone and ethanol extracts of the leaves, bark, and roots of *C. angolense* exhibit antioxidant activities comparable to those of standard antioxidants such as quercetin and curcumin. This finding is supported by literature that also emphasizes the effectiveness of ethanolic and acetonic extracts in neutralizing free radicals [45,46,47,48].

The antioxidant activity observed reflects the presence of a diverse array of bioactive phytochemicals in these extracts. Predominantly, polyphenols—including phenols and flavonoids—were abundant, along with other classes such as alkaloids, quinones, terpenes, and aldehydes. These compounds are believed to play a crucial role in mitigating oxidative stress, thereby providing a consistent result for their health-promoting properties [31]. Several studies on *C. angolense* have consistently identified flavonoids and phenols as the predominant bioactive components [20,49]. For instance, research on the aqueous extract of both fresh and dried bark of *C. angolense* confirmed high levels of flavonoids and phenols with strong antioxidant activity, which supports the results obtained from the leaves, bark, and roots in our study [20].

Moreover, the chemical composition and corresponding antioxidant activities of the extracts are influenced by variations in the bioactive phytochemical content inherent to medicinal plants [38,50,51]. Natural antioxidants in plants are known to inhibit free radical chain reactions by interfering with both the initiation and propagation stages, ultimately leading to chain termination and slowing down the overall oxidation process [21,52,53].

Additional studies have revealed that aqueous extracts of the roots of *C. angolense* contain bioactive substances with antiviral properties and the ability to inhibit the growth of *Plasmodium falciparum* and *Plasmodium berghei* in vitro [54,55,56]. The identification of various bioactive phytochemicals—including flavonoids, phenols, terpenes, and alkaloids—in our study aligns with these findings and supports previous reports. Preliminary investigations on the aqueous extracts of the roots and bark have also confirmed high concentrations of antioxidant compounds, directly correlating with their performance in the antioxidant assays [11].

The diversity of phytochemical compounds found in the leaves, bark, and roots of *C. angolense* underscores the significance of this plant as a source of various bioactive substances. The chemical composition of its polyphenolic compounds can vary compared to other species in the same family and is influenced by factors such as climatic conditions, harvest season, soil quality, and the processing and analytical methods used for identification. These variables contribute to the wide range of values reported in different studies [57,58,59].

Differences in the bioactive phytochemicals identified in our results and those reported in the literature can also be attributed to variations in extraction methods, as well as the geographic regions and seasons of harvest. According to [58,60], one of the main challenges in assessing the antioxidant activity of biological materials is the choice of analytical method, as each assay tends to target a specific aspect of antioxidant activity. Nonetheless, the four assays we performed yielded consistent results across the evaluated extracts, further confirming that *C. angolense* is a rich source of antioxidants.

Some studies have reported superior antioxidant activity in methanolic, hydromethanolic, and infusion extracts of *C. angolense* collected in southern Angola [39,49,60]. It is suggested that environmental factors—such as climatic conditions, soil types, and the use of relatively more polar solvents—can enhance antioxidant activity. In general, factors that influence the content of secondary metabolites in medicinal plants indicate a positive correlation between solar radiation intensity and the production of phenolic compounds, including flavonoids, tannins, and anthocyanins. However, the specific climatic conditions, time of year, or regions under which these correlations hold true are not always clearly defined, making comprehensive comparisons challenging [26].

The antioxidant activities of the leaves, bark, and roots, as demonstrated by our assays, align with the variations reported in the literature and reflect the broad diversity of bioactive compounds identified in this study [24,61,62]. The high antioxidant capacity observed in our tests correlates positively with the presence of phytochemicals such as flavonoids, phenols, alkaloids, and terpenes. Several studies have documented the relationship between antioxidant activity and polyphenolic content [40].

Moreover, numerous studies have employed various organic solvents for the extraction of compounds from different parts of medicinal plants in the Cochlospermaceae family and other medicinal plant families. Many of these findings support our results [11,60,63,64,65]. For instance, the methanolic extract of the root of *C. planchonii* has been shown to contain bioactive compounds with potent pharmacological activities in rats and low toxicity, primarily attributed to flavonoids and phenols—compounds also identified in our study [66].

The phytochemical characterization of extracts from the leaves, bark, and roots—as well as the fractions of the hydroethanolic and ethyl acetate extracts—from *C. regium* (Schrank) confirmed the presence of flavonoids and phenols, both of which are known for their antioxidant and antimicrobial activities. These findings further validated the flavonoid and phenol profiles identified in our study [36,52]. For instance, research on the leaves and stem bark of *C. gossypium* has documented numerous bioactive constituents, including flavonoids and phenolic compounds with notable antibacterial and antioxidant properties, and has specifically reported the antioxidant activity of the methanolic leaf extract [40]. Additionally, the biological activities of aqueous methanol extracts from the leaves, roots, and root bark of *C. tinctorium*—where flavonoids are also present—have been well documented [67].

Considering the diverse array of bioactive phytochemicals reported in previous research on various species within the Cochlospermaceae family, it is evident that *C. angolense* is a rich source of compounds with distinct pharmacological properties. The different bioactive polyphenolic compounds, particularly flavonoids and phenols, have attracted significant interest due to their potential benefits for human health. The chemistry and biology of these compounds are critical to understanding their biological effects [68,69].

Among the flavonoids most reported in the literature on extracts from different parts of the plant are querctrin, naringenin, astragalin, vitexin, apigetrin, pinobanksin, quercetin-3′-glucuronide, isoquercetin, morin, quercetin-3-arabinoside, dihydroisorhamnetin, kaempferol, and (+)-dihydrokaempferol. Notably, dihydrogenistein (found in the bark and roots), (−)-8-prenylnaringenin (present in the leaves and roots), isoquercetin (detected in the bark), and pinobanksin (identified in the leaves and roots) are reported for the first time in *C. angolense*. Isoquercetin is a glycosylated form of quercetin, the main flavonoid present in the human diet, widely studied for its therapeutic properties. Its main biological activities include antioxidant, anti-inflammatory, antiviral, antitumor, and cardioprotective [70]. Pinobanksin is a flavonol, belonging to the flavonoid class, recognized for its biological properties, including antioxidant, antimicrobial, and anti-inflammatory activities. This compound is commonly found in Lavandula stoechas [30,71]. It has been reported that Pinobanksin, one of the propolis compounds, is also found in some plant species. Propolis compounds have shown inhibitory effects against the ACE2, TMPRSS2, and PAK1 signaling pathways of SARS-CoV-2 in studies. Similarly, isoquercetin is structurally similar to quercitrin, which is currently in clinical trials as a potential drug for COVID-19 [72].

Quercetin, a well-known flavonol, has a longstanding history in folk medicine for treating colds, flu, and rheumatic conditions, and as a diuretic. It is a potent antioxidant with significant biological, pharmacological, and medicinal properties, including the inhibition of human platelet aggregation in vitro [34,64,73,74,75,76]. Additionally, quercetin exhibits potential anticancer effects by inducing cellular differentiation and, in its glycosidic form, inhibiting protein tyrosine kinase [69,75]. Quercitrin, a glycoside of quercetin, has been reported in the methanolic extract of *Alchornea cordifolia* leaves, where it demonstrated anti-inflammatory properties [77].

Similarly, apigetrin (apigenin-7-glucoside) is a glycosidic flavonoid with notable antioxidant potential. It is found in several medicinal plants, such as *Tricholaena teneriffae* [76], *chamomile* [78], and *Scutellaria baicalensis* [79]. Moreover, the ethanolic extract of Bauhinia hookeri has revealed that flavonoids like kaempferol and quercetin possess multiple biological effects, including robust antioxidant activity [57,74,79,80]. These compounds help prevent oxidative stress by directly scavenging free radicals, chelating metals, and inducing antioxidant enzyme expression.

In addition, quercetin, as identified in *Piliostigma thonningii* (Schum), has been documented to possess not only strong antioxidant activity but also a wide range of pharmacological properties, including anticancer, antiviral, anti-obesity, antifungal, antibacterial, and antidiabetic effects [64]. In a separate study, the 70% methanolic extract of the leaves and roots of *Ximenia caffra* from Western and Eastern Sub-Saharan Africa led to the identification of quercetin, further confirming its potent antioxidant capacity [24,81].

Data obtained from the hydroethanolic extract of *C. regium* (Mart. ex Schrank) revealed the presence of both morin and kaempferol [14]. Kaempferol, a flavonol and a derivative of flavonoids, has demonstrated excellent free radical scavenging abilities in DPPH assays and effectively inhibits lipid peroxidation in human low-density lipoproteins as measured by the TBARS assay [82]. Widely found in plant-based foods such as kale, beans, tea, spinach, and broccoli, kaempferol is recognized for its powerful antioxidant properties as well as its anti-inflammatory, anticancer, and cardioprotective effects [74]. Moreover, kaempferol has shown hepatoprotective properties in models of liver injury induced by CCl_4_, various drugs, and alcohol, suggesting its potential as a therapeutic agent for patients with atherosclerotic disease [17]. Additionally, flavonols such as rutin, morin, myricetin, and kaempferol have been reported to exert antisecretory and cytoprotective effects in animal models of gastric ulcer, as well as anti-*Helicobacter pylori*, anti-ulcer, antioxidant, and anti-inflammatory activities [14].

Vitexin (apigenin-8-c-beta-d-glucopyranoside) is another important flavonoid derivative—a glycoside of apigenin—that exhibits a broad spectrum of biological activities, including antioxidant, antimicrobial, anti-inflammatory, neuroprotective, anticancer, antiviral, antihypertensive, antiasthmatic, and antiepileptic effects [83]. This phytochemical is found in several plants, such as passionflower (*Passiflora*), bamboo (*Phyllostachys nigra*), pearl millet (*Pennisetum glaucum*), and hawthorn (*Crataegus*) [84]. Notably, vitexin has been shown to offer protective effects in the intestines by modulating macrophage polarization in azoxymethane-treated mice [85].

Quercetin-3-glucuronide, a flavonoid present in wine, fruits, and medicinal herbs, is recognized as the main circulating metabolite in human plasma following the consumption of quercetin-rich foods. It exhibits a range of beneficial activities, including antioxidant, anti-inflammatory, antiviral, anticancer, and neuroprotective effects [85]. Given these promising attributes, quercetin-3-glucuronide is considered a potent phytochemical for disease prevention and health promotion. Indeed, both quercetin and its glucuronide derivative rank among the most powerful antioxidants available from natural sources [70].

Morin (2′,3,4′,5,7-pentahydroxyflavone) is a flavonol derived from the flavonoid family, commonly found in fruits and vegetables such as onions, apples, and tea [86,87]. This phytochemical offers promising health benefits, including antioxidant, anti-inflammatory, and anticancer activities [20]. Research has shown that morin can protect against oxidative stress and reduce inflammation, which may be beneficial in preventing and managing various chronic diseases [14].

Licochalcone, also known as 3′-prenylnaringenin, is a flavanone derivative found in Glycyrrhiza glabra. It exhibits significant antioxidant, antimicrobial, and anticancer properties [88]. Notably, licochalcone has been observed to inhibit the energy metabolism of breast cancer cells, thereby reducing tumor proliferation, invasiveness, and stemness [89].

Naringenin, primarily found in citrus fruits and rosemary [90], is recognized as a bioactive compound with strong antioxidant properties and multiple health benefits. This phytochemical has been identified in methanolic extracts of the leaves, root bark, and wood of *C. vitifolium* [91,92], as well as in ethanolic extracts of the root bark, wood, flowers, and stems of *C. gillivraei* [17]. Previous studies indicate that naringenin can help prevent oxidative stress and inflammation in liver injuries, act as a potent lipid-lowering agent, and protect against obesity and glucose intolerance in rat models [93,94]. Moreover, it has been shown to reduce blood pressure in rats, suggesting that naringenin may help safeguard liver function by mitigating oxidative and inflammatory damage associated with liver injuries [91,95].

(+)-Dihydrokaempferol, also known as aromadendrin, is a flavonoid found in various plants and medicinal herbs [82,96]. This compound exhibits a broad range of pharmacological activities, including potent antioxidant, anti-inflammatory, and antidiabetic effects [73,97]. Aromadendrin has been identified in extracts from the bark and roots of *C. vitifolium* [17,98].

Pinobanksin is recognized for its diverse biological properties, such as antioxidant, antimicrobial, and anti-inflammatory activities, and is commonly found in *Lavandula stoechas* [21,71].

(−)-8-Prenylnaringenin (8-prenylnaringenin) is a powerful phytoestrogen primarily found in hops (*Humulus lupulus*) [99]. Celebrated for its wide range of biological activities and significant health benefits, this compound is among the most potent phytoestrogens and is used to alleviate menopausal and postmenopausal symptoms associated with declining hormonal levels in women [100,101].

Morachalcone, a type of chalcone, is present in several medicinal plants, including *Glycyrrhiza glabra* [102], *Campomanesia adamantium* [103], *Cochlospermum tinctorium* [104], *and Sorbus commixta* Hedl [105]. Known for its remarkable biological properties, morachalcone has demonstrated significant antioxidant, antibacterial, antifungal, antiviral, and anticancer activities in various studies [16,46,106].

The phenolic compounds identified in the extracts under study demonstrate significant antioxidant properties, protecting cells from oxidative damage [107]. Numerous studies have confirmed the antioxidant potential of these phenolic phytochemicals and phenolic acids, and our findings further underscore their importance in *C. angolense* [108,109,110].

Embelin (2,5-dihydroxy-3-undecyl-1,4-benzoquinone), a natural compound derived from Embelia ribes, exhibits a wide range of pharmacological activities, which are partly attributed to its ability to scavenge free radicals [111,112]. Its therapeutic spectrum includes antioxidant, anti-inflammatory, anticonvulsant, antifertility, anti-implantation, hepatoprotective, analgesic, wound-healing, and antibacterial effects [113,114]. Moreover, embelin has been reported as an inhibitor of cancer cell growth, making it a promising anticancer candidate despite its limited clinical applicability due to low water solubility [115].

Antiarol (3,4,5-trimethoxyphenol) is another natural compound, isolated from *Cochlospermum vitifolium*, *Antiaris toxicaria*, and *Mentha aquatica*, known for its antioxidant properties and potential applications in various biological and pharmaceutical contexts [17,30,116].

Zingerone (vanillylacetone) is recognized for its antioxidant and antidiarrheal properties [117]. It effectively scavenges free radicals and reactive oxygen species, and inhibits enzymes involved in their production [118]. Similarly, (+)-[6]-gingerol, a major bioactive constituent of ginger, is celebrated for its diverse biological activities—including antioxidant, anti-inflammatory, antimicrobial, anticancer, neuroprotective, cardiovascular and respiratory protective, anti-obesity, antidiabetic, anti-nausea, and antiemetic effects [109,119]. This phytochemical has also been identified in the lichen *Parmotrema hypoleucinum* from Algeria [120], highlighting its value as a dietary molecule with significant health benefits, particularly in tumor treatment and the inhibition of liver and gallbladder inflammation [121].

Oleuropein, which imparts the characteristic bitter taste to olives and is the primary contributor to the anti-inflammatory activity of olive leaf extract, is one of the most extensively studied compounds. Along with its derivative hydroxytyrosol, oleuropein is known for its potent antioxidant properties and a wide array of health benefits, including anti-inflammatory, anti-atherogenic, anticancer, antimicrobial, antiviral, and protective effects against cardiovascular and metabolic diseases [122,123,124]. A recent study on *Lavandula pedunculata* reported the presence of oleuropein and demonstrated its antioxidant, anti-inflammatory, and antimicrobial activities [21].

Coumarandione (dicoumarol) is a phenol-derived coumarin, a secondary metabolite present in various plants such as guaco and fennel [107]. This natural compound functions as an anticoagulant and was historically significant as the first oral drug used to prevent blood clot formation. In addition to its anticoagulant properties, dicoumarol and other coumarins exhibit antioxidant activity and can act as enzyme inhibitors [3,48].

Herniarin, another phenol-derived coumarin naturally occurring in certain plant species [8], also displays antioxidant properties. It has been shown to reduce radiation-induced cytotoxicity and genotoxicity in human lymphocytes [125]. Identified in the aqueous extract of *Glycyrrhiza glabra*, herniarin has been used therapeutically to treat hepatitis by lowering liver transaminase levels [102]. Moreover, German chamomile flowers, particularly the late liguliflorae, have been reported to contain both herniarin and umbelliferone (7-hydroxycoumarin), which may contribute to chamomile’s allergenic potential [46,126]. Herniarin is also being explored as a potential chemoprotective agent against cisplatin-induced genotoxicity [127].

The presence of these bioactive phytochemicals in the studied extracts may help explain the traditional use of this plant against hepatitis in the Cuanza Norte Province [128]. In general, phytochemicals such as flavonoids and phenolics are known for their antioxidant activity, which helps protect cells from oxidative damage caused by free radicals. Increasing attention is being given to these compounds for their potential to counteract the harmful effects of free radicals and mitigate chronic diseases associated with aging [62,69,129].

In addition to flavonoids and phenolics, other classes of bioactive phytochemicals identified in this study—including alkaloids, terpenes, tannins, and quinones—exhibit significant antioxidant properties [66]. Alkaloids, which play a crucial role in plant defense against insects and herbivores, are also highly valued in medicine for their therapeutic benefits. Well-known examples include morphine and nicotine [130]. For instance, elaeococanin C, an alkaloid reported in the leaves of *Elaeocarpus kalziensis* Schltr, demonstrates notable biological activity and is also present in *Averrhoa bilimbi* [30,131].

Similarly, evocarpine, a quinolone alkaloid primarily isolated from the fruit and herb of *Evodia rutaecarpa*, exhibits slight toxicity and has shown a pronounced antagonistic effect against various mycobacterial strains [132,133]. Hygrine, a ketonic alkaloid naturally occurring in coca leaves (*Erythroxylum coca*) and other plants, is of considerable interest due to its mydriatic action [134]. Additionally, edpetiline, a natural alkaloid found in *Petilium eduardi*, is noted for its anti-inflammatory and antioxidant properties and is predominantly used in research settings [135].

Terpenes are renowned for their aromatic properties and are widely used in the perfume, cosmetics, food, and pharmaceutical industries. They also fulfill important ecological roles, such as attracting pollinators or repelling herbivores [136]. Ganoderic acid, a triterpenoid derived from lanosterol and found in *Ganoderma lucidum* and other Ganoderma species, displays a range of pharmacological activities including anticancer, antiviral, antioxidant, and platelet aggregation inhibition properties [137]. These compounds have been extensively studied in traditional medicine for their potential health benefits, with some studies reporting anti-HIV-1 activity, hepatoprotective, antihypertensive, and cholesterol-lowering effects [138,139,140].

Umbellatolide B, a lactone and monoterpenoid classified as an 11-noriridoid, is another notable phytochemical present in various plants. Isolated from *Morinda umbellata*, it has been investigated for its potential antimicrobial and anti-inflammatory activities, and is traditionally used to prevent and treat inflammation, cancer, neurological disorders, and age-related conditions [141,142].

Farnesylacetone is a terpenic ketone renowned for its anti-inflammatory and antifungal properties, which underlie its frequent use in traditional medicine and cosmetic products for therapeutic benefits Pterosin B, a sesquiterpenoid found in ferns such as *Pteridium aquilinum*, has attracted research interest due to its intriguing pharmacological properties and its effects on chondrogenic cells [143,144].

Lucialdehyde B is a triterpene extracted from the fruiting bodies of certain plants; it exhibits notable cytotoxic activity, with studies focusing on its potential against cancers such as breast cancer and sarcoma. In *C. angolense*, this compound, identified in the bark, has demonstrated antiviral and cytotoxic effects against various cancer cell lines [145].

Farnesolic acid is another natural terpenoid present in diverse sources, including the leaves of *Myrcia alagoensis* (Myrtaceae) and even marine organisms like crustaceans. It serves as an important precursor in the biosynthesis of hormones and other bioactive compounds [146]. Beyond its role in insect juvenile hormone biosynthesis [147], farnesolic acid exhibits antimicrobial, anti-inflammatory, and potential antitumor properties by modulating various biological pathways [135,148].

Urechitol B, a natural terpenoid found in the root extracts of *Pentalinon andrieuxii*—a plant used in traditional Mayan medicine—is influenced by the flowering stage, which affects its production [149,150].

Loliolide, a ubiquitous monoterpenoid lactone first described in 1974, has been isolated from both plants and animals. It is also present in several marine brown algae such as *Sargassum horneri*, *Sargassum ringgoldianum subsp. coreanum*, and *Undaria pinnatifida*, where it exhibits antioxidant, antifungal, antibacterial, and anticancer activities [151]. In the medicinal plant *Lactuca serriola*, loliolide has been associated with a range of biological effects, including hepatoprotection, antioxidant, antivenom, allelopathic, sedative, anticonvulsant, antiepileptic, anti-inflammatory, and anticancer activities [30].

Ellagic acid rhamnoside is the sole tannin identified in the ethanolic extract of the bark, noted for its antimicrobial activity [152]. Research on ellagic acid and its derivatives from various plant species has revealed a spectrum of pharmacological properties, including antioxidant, antimicrobial, anticancer, antidepressant, cardiovascular protective, antidiabetic, neuroprotective, and anti-inflammatory effects [153].

Our investigation also uncovered additional bioactive phytochemicals with diverse pharmacological activities that fall outside the previously mentioned classes. Ostopanic acid, also known as (*E*,*E*)-7,12-dioxooctadeca-8,10-dienoic acid, is a cytotoxic fatty acid initially isolated from the stems and fruits of *Ostodes paniculata* and later from the hallucinogenic mushroom *Gymnopilus spectabilis* [154]. This compound has demonstrated potential in inhibiting the growth of certain cancer cells, such as P-388 lymphocytic leukemia cells [129,155].

Sorbic acid (2,4-hexadienoic acid) is a natural organic compound found in the fruits and seeds of *Sorbus aucuparia* [38]. Primarily employed as a preservative in the food and beverage industry, it effectively inhibits bacteria, yeasts, and fungi by reducing their capacity for amino acid absorption. Notably, the literature indicates that sorbic acid poses minimal health risks and is not associated with cancer; it was first isolated in 1859 from the unripe fruits of the rowan tree (*Sorbus aucuparia*), which explains its name [155].

Valerenic acid, identified in the studied leaves, is a major component of the volatile oils and roots of the medicinal plant *Valeriana officinalis*. This compound is renowned for its diverse pharmacological activities, particularly its sedative and anxiolytic properties, which make it a key ingredient in natural treatments for insomnia and anxiety [8,156].

Cinnamaldehyde, a natural compound found in cinnamon, exhibits a range of biological activities, including antimicrobial, anti-inflammatory, angiogenic, and wound-healing effects [110]. Widely used as a spice and in traditional herbal medicine, cinnamon (*Cinnamomum* species) owes much of its biological efficacy to cinnamaldehyde, the principal active component of its essential oil. This compound has been associated with antimicrobial, antioxidant, and antidiabetic activities [28,157]. Furthermore, several studies indicate that when cinnamaldehyde is combined with other phytochemicals such as eugenol, thymol, and carvacrol, it exhibits enhanced, synergistic biological effects [158,159]. In our study, this compound was detected in the leaves and bark of *C. angolense* and has also been identified in the root oils of *C. angolense* as well as in the fruit oils of *Alchornea cordifolia* [116,160].

Benzaldehyde is another naturally occurring product with significant biological activity. Commonly used as a flavoring and fragrance agent in foods, perfumes, and the dye industry, benzaldehyde also exhibits notable pharmacological properties and has even been described as a bee repellent [12,161].

Studies on C. angolense extracts have shown that Borututu root contains high levels of antioxidant compounds, directly contributing to its pharmacological potential. These findings can be correlated with previous studies on bioactive phytochemicals identified in medicinal plants of the same species and other related species, emphasizing their significant antioxidant activities in the acetone and ethanol extracts of the leaves, bark, and roots [162,163,164,165].

Ultimately, our findings underscore that numerous phytochemicals present in the analyzed extracts exhibit antioxidant activity comparable to quercetin and curcumin. These bioactive compounds not only neutralize free radicals—thereby safeguarding cells and tissues from oxidative damage—but also contribute to the prevention of various chronic diseases [166,167,168]. This robust antioxidant capacity likely underpins the plant’s longstanding traditional use among rural communities in Angola, particularly in the Cuanza Norte region, where its therapeutic benefits are highly valued. Furthermore, our results pave the way for future research into the development of novel, plant-based therapeutic agents that capitalize on these bioactive properties.

## 3. Materials and Methods

### 3.1. Sample Collection and Authentication

**Figure 2:**
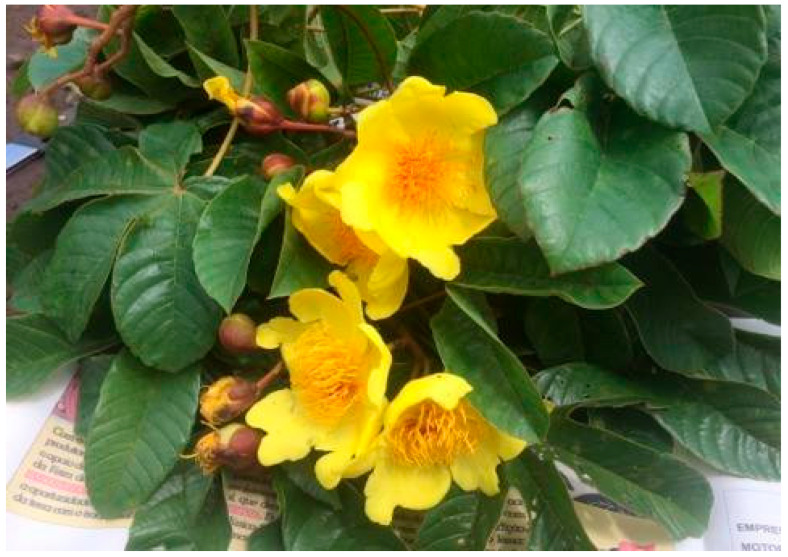
Angolan *Cochlospermum angolense* Welw ex Oliv.

**Figure 3:**
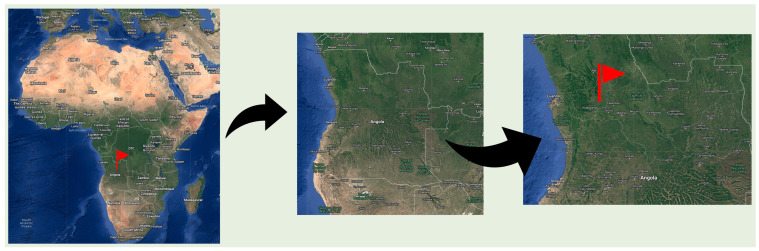
The topography of Angola, indicating provincial boundaries and region of study.

Roots, barks, and leaves of *Cochlospermum angolense* Welw ex Oliv were collected in March 2017 in the Kwanza Norte Province, near Ndalatando, Angola, with the consent of local traditional authorities and in accordance with the United Nations Convention on Biodiversity (Figure 2). All samples were gathered during the flowering season (coordinates S9°16.493′, E15°01.203′; altitude: 791 m) (Figure 3). The species was authenticated by botanist Christin Heinze from the University of Dresden, Germany (Vaucher DR 044693). The samples were dried in a location shielded from direct sunlight and stored in sealed bags. A reference sample from each was subsequently deposited at the Laboratory of Fiber Materials and Environmental Technologies (FibEnTech), University of Beira Interior, Covilhã, Portugal.

### 3.2. Chemicals and Reagents

All chemicals and reagents used in this study were of analytical grade. These included n-hexane, acetone, ethanol, chloroform, ethyl acetate, curcumin, quercetin, dimethyl sulfoxide (DMSO), 2,2-diphenyl-1-picrylhydrazyl (DPPH), sodium nitroprusside dihydrate, phosphoric acid, sulfanilamide, N-(1-naphthyl)ethylenediamine dihydrochloride, sodium phosphate dibasic, potassium chloride, nitro blue tetrazolium dihydrochloride, *β*-nicotinamide adenine dinucleotide (NADH), phenazine methosulfate, 2-thiobarbituric acid, ferrous sulfate heptahydrate, trichloroacetic acid (TCA), and sodium lauryl sulfate (SDS). All reagents were purchased from Sigma-Aldrich, Steinheim, Germany.

### 3.3. Samples Preparation and Extraction

Organic hexane extracts were first obtained via Soxhlet extraction. Specifically, 308.97 g of leaves, 544.31 g of bark, and 720.82 g of roots from *Cochlospermum angolense* were extracted over a 24-h period using approximately 2500 mL of n-hexane. Following extraction, the samples were filtered, and the solvent was removed under vacuum using a rotary evaporator set at 35 °C.

After hexane extraction, the residual plant materials were transferred into 20-L flasks, and acetone was added at a ratio of 25 g of plant material to 100 mL of acetone. The extraction was allowed to proceed for one week. The resulting acetone extracts were then filtered and evaporated under vacuum in a rotary evaporator at 40 °C until dry.

Subsequently, the same plant materials were subjected to ethanol extraction under similar conditions. Ethanol was added at a ratio of 25 g of plant material to 100 mL of ethanol, and the extraction was maintained for one week. After filtration, the ethanol extracts were evaporated under vacuum in a rotary evaporator at 40 °C until dryness.

All organic extracts were then weighed, and the different masses along with their respective yields (calculated by comparing the mass of the extracts to the initial mass of the plant material) are presented in Table 3.

### 3.4. Evaluation of Antioxidant Activity of the Extracts

#### 3.4.1. 2,2-Diphenyl-1-picrylhydrazil Radical (DPPH^•^)-Scavenging Activity

The determination of the DPPH radical inhibition percentage was carried out following the methodology proposed by [169,170]. In each well of a 96-well microplate, 100 µL of DPPH and 100 µL of extracts, previously dissolved in DMSO, was added at concentrations ranging from 0.48 to 125 µg/mL. Quercetin was used as positive control. The microplate was incubated in the dark at room temperature for 30 min, and the absorbance was measured at a wavelength of 492 nm using an microplate reader (Promega Glo-max^®^-Multi+ reader, model E9032, Promega, Madison, WI, USA). All experiments were performed in triplicate. The percentage of DPPH radical scavenging was calculated using Equation (1):

(1) $$Free\;radical\;scavenging\;\left(\%\right)=\left[A_{control}-A_{test}\right]\times 100/A_{control}$$

where A_control_ is the absorbance of the reaction media without the test sample and A_test_ is the absorbance in presence of the essential oil or Quercetin. The IC_50_ values—the concentration of compound required to reduce the DPPH absorbance by 50%—were determined by linear regression analysis of the dose-response data.

#### 3.4.2. Nitric Oxide Radical Assay

The nitric oxide (NO) scavenging activity was determined using a Griess Illosvoy reaction-based assay [171]. Briefly, 50 μL of the extract, 50 μL of a 10 mM sodium nitroprusside solution, and 50 μL of Griess reagent prepared in phosphate-buffered saline (PBS, pH 7.4) were mixed in a 96-well microplate. The extracts were previously dissolved in DMSO and tested at concentrations ranging from 0.48 to 125 μg/mL. The plate was incubated at 25 °C for 90 min, after which the absorbance was measured at 560 nm.

All measurements were performed in triplicate. The NO radical scavenging activity (%) was calculated using Formula (1).

#### 3.4.3. Superoxide Radical Assay

The superoxide anion (O_2_•^−^) scavenging activity of extracts was determined using an NADH-PMS system, following the method described by [49], with slight modifications. The assay was conducted at various concentrations of extract or quercetin (0.48 to 125 µg/mL), and absorbance readings were taken at 560 nm using Promega Glo-max^®^-Multi+ reader (model E9032). The IC_50_ values obtained were then compared to those for quercetin. All samples were tested in triplicate. The percentage of O_2_•^−^ activity was calculated using the same formula applied for the DPPH radical inhibition assay.

#### 3.4.4. Lipid Peroxidation Inhibition Assay

A modified thiobarbituric acid reactive substances (TBARS) assay [172] was employed to measure lipid peroxidation, using egg yolk homogenates as the lipid-rich medium. In this adapted method, 100 μL of egg homogenate (diluted 1:25 *v/v* in PBS, pH 7.4) and 10 μL of extract or positive control (curcumin) at various concentrations (0.48 to 125 µg/mL) were added to a test tube along with 25 mmol/L of freshly prepared homogenate. The mixture was incubated at 37 °C for 15 min. The reaction was terminated by adding 50 μL of 15% (*w*/*v*) trichloroacetic acid (TCA), followed by centrifugation at 3500 rpm for 15 min. A 200 μL aliquot of the supernatant was mixed with 100 μL of 0.8% thiobarbituric acid (TBA) in 1.1% sodium dodecyl sulfate (SDS) and heated in a water bath at 95 °C for 30 min. After cooling, the absorbance of each sample was measured at 532 nm to quantify the MDA levels. The percentage inhibition of lipid peroxidation was calculated using Equation (2).

(2) $$Inhibition\;of\;lipoperoxidantion\;\left(\%\right)=\left[A_{control}-A_{test}\right]\times 100/A_{control}$$

where A_control_ is the absorbance of an egg yolk emulsion in a blank buffer without the test sample and A_test_ is the absorbance of the egg yolk emulsion containing either the extracts or the standard substance (curcumin).

### 3.5. Profiling of Chemical Compounds by High-Performance Liquid Chromatography with Electrospray Ionization and Tandem Mass Spectrometry Detection (HPLC-ESI-MS-MS)

The analysis of phenolic compounds, including flavonoids (e.g., flavanones, glycosides, and methoxyflavones), coumarins, and phenols, as well as non-phenolic compounds such as alkaloids, terpenoids (acyclic monoterpenoids and sesquiterpenes), polyketides, benzoquinones, aromatic esters, and ascorbic acid derivatives, was carried out according to the method of Lahlou et al. [30], on an or-bitrap Thermo q-Exactive (Thermo Fisher Scientific, Waltham, MA, USA) mass spectrometer coupled to a Vanquish HPLC. A Kinetex XB-C18 (Phenomenex, Aschaffenburg, Germany) with a particle size of 2.6 microns, 100 mm in length, and a diameter of 2.1 mm was used as a column. The mobile phases were 0.1% formic aqueous solution (A) and acetonitrile (B). The gradient program (time (min), % B) was as follows: (0.00, 50); (20.00, 100); (25.00, 100); (35.00, 50). The flow rate was 0.200 mL min^−1^, and the injection volume was 10 µL. The ionization electrospray in positive mode was used. The following analysis parameters were electrospray voltage, −3.8 kV, sheath gas flow rate, 30; auxiliary gas unit flow rate, 10; drying gas temperature, 310 °C; capillary temperature, 320 °C; S-lens and RF level, 55. The acquisition was performed in a mass range from 100 to 1000 a.m.u. An auto MS2 program was used with a fragmentation voltage of 30 V.

### 3.6. Statistical Analysis

All determinations were made in triplicate, and values were expressed as mean ± standard deviation (SD). Statistical differences were determined by analysis of variance (ANOVA) and the least significant difference (LSD) test, with a *p* < 0.05 for comparison of means in each of the variables analyzed in the different trials. All statistical analyses were performed with GraphPad 9.0 software.

## 4. Conclusions

*Cochlospermum angolense* Welw ex Oliv contains a broad array of bioactive phytochemicals—including flavonoids, phenols, sesquiterpenoids, quinones, and alkaloids—that underpin the significant antioxidant activity observed in this study. These compounds play a crucial role in neutralizing free radicals and alleviating oxidative stress, thereby supporting the plant’s traditional use in Angolan medicine. In practice, various formulations such as infusions, pills, and syrups made from the leaves, bark, and roots are employed to harness these antioxidant, antimicrobial, and anti-inflammatory properties, particularly in the treatment of liver diseases.

Our research further expands the phytochemical profile of *C. angolense* by identifying several novel compounds, including (+)-abscisic acid, angustine B, pinobanksin, dihydrogenistein, (−)-8-prenylnaringenin, isoquercetin, samandarine, dihydromyricetin, and eupatoriocromene. The discovery of these compounds not only enhances our understanding of the plant’s chemical complexity but also suggests potential new avenues for therapeutic application. The high concentrations of flavonoids, phenols, and terpenes contribute to its robust antioxidant capacity, making *C. angolense* a promising candidate for development into conventional pharmaceutical agents.

Moreover, with this work, the use of traditional medicinal knowledge is complemented with scientific research results, providing a strong base for further research. The potent bioactivities observed in our extracts provide a compelling rationale for future studies. The objectives were accomplished; nevertheless, in future work, some topics may be addressed. Studies using in vivo models, and comprehensive toxicity testing, along with the optimal dosage for effectiveness, may be established for the extrapolation of the results to therapeutic applications. The exact mechanisms of action of the active compounds also remain unknown, as do the possible synergistic or antagonistic interactions among the phytochemicals present in the extracts. Future research should focus on exploring in vivo studies, toxicity, and the synergistic interactions among these phytochemicals, as well as their potential roles in the prevention or mitigation of chronic diseases associated with oxidative stress. Overall, our study lays a solid foundation for the continued exploration of *C. angolense* as a valuable resource for novel therapeutic agents in conventional medicine.

## Figures and Tables

**Table 1:** Phytochemical characterization of bioactive constituents present in acetone and ethanol extracts obtained from leaves, bark, and roots of *C. angolense*.

RT (min)	Compound	[M + H]^+^	MW Calc.	Formula	Frags +	Frags +	Frags +	Frags +	Leaves		Barks		Roots	
									(acetone)	(ethanol)	(acetone)	(ethanol)	(acetone)	(ethanol)
7.73	(+)-Abscisic acid	265.1432	264.1354	C_15_H_20_O_4_	91.0545	105.0701	79.0547	119.0854	-	-	-	-	Detected	-
7.95	Loliolide	197.1171	196.1093	C_11_H_16_O_3_	91.0545	65.0392	105.0701	79.0545	-	-	-	-	Detected	-
7.97	Quercitrin	449.1073	448.0995	C_21_H_20_O_11_	329.0648	299.0545	353.0644	413.0858	-	Detected	-	-	-	-
7.98	Samandarin	306.2460	305.2382	C_19_H_31_NO_2_	100.1123	55.0549	127.0755	182.1537	Detected	-	-	-	-	-
8.10	Astragalin	449.1072	448.0994	C_21_H_20_O_11_	299.0547	329.0652	353.0649	325.0698	-	Detected	-	-	-	-
8.10	Eupatoriochromene	219.1013	218.0935	C_13_H_14_O_3_	115.0542	128.0620	91.0545	174.0670	-	-	Detected	Detected	-	-
8.20	Vitexin	433.1125	432.1047	C_21_H_20_O_10_	283.0596	313.0699	121.0284	297.0747	-	Detected	-	-	-	-
8.38	Apigetrin	433.1122	432.1044	C_21_H_20_O_10_	283.0594	313.0699	337.0698	165.0179	-	Detected	-	-	-	-
8.40	Dihydromyricetin	321.06	320.0522	C_15_H_12_O_8_	163.0386	219.0284	191.0335	107.0492	-	-	-	-	Detected	-
8.47	Hydroconiferyl Alcohol	183.1014	182.0936	C_10_H_14_O_3_	95.0494	51.0236	91.0546	115.0542	-	Detected	-	-	-	-
8.47	Naringenin	273.0752	272.0674	C_15_H_12_O_5_	153.0179	91.0544	68.9975	119.0491	-	-	Detected	Detected	-	-
8.55	Quercetin-3′-glucuronide	479.0813	478.0735	C_21_H_18_O_13_	303.0493	85.0287	153.0180	229.0490	-	Detected	-	-	-	-
8.58	Zingerone	195.1014	194.0936	C_11_H_14_O_3_	91.0542	95.0494	79.0547	53.0392	-		-	-	Detected	-
8.60	Isoquercetin	465.1023	464.0945	C_21_H_20_O_12_	303.0493	85.0288	229.0490	137.0231	-	Detected	-	-	-	-
8.62	(+)-Dihydrokaempferol	289.0703	288.0625	C_15_H_12_O_6_	107.0493	153.0179	149.0231	215.0698	-	-	Detected	-	-	-
8.62	Dihydroisorhamnetin	319.0809	318.0731	C_16_H_14_O_7_	137.0596	153.0181	149.0232	122.0364	-	-	-	-	Detected	-
8.64	Pterosin B	219.1377	218.1299	C_14_H_18_O_2_	121.0648	91.0545	107.0493	149.0595	-	-	-	-	Detected	Detected
8.70	Morin	303.0495	302.0417	C_15_H_10_O_7_	153.0179	229.0494	137.0233	68.9976	-	Detected	-	-	-	-
8.73	Quercetin-3-Arabinoside	435.0916	434.0838	C_20_H_18_O_11_	303.0493	73.0289	153.0179	229.0485	-	Detected	-	-	-	-
8.90	Evocarpine	340.2590	339.2512	C_23_H_33_NO	114.0914	96.0810	69.0704	209.1645	-		-	-	-	Detected
8.95	Kaempferol	287.0545	286.0467	C_15_H_10_O_6_	153.0180	121.0284	68.9976	213.0541	-	Detected	-	-	-	-
8.98	Cinnamaldehyde	133.0648	132.0570	C_9_H_8_O	51.0236	95.0494	115.0541	91.0546	Detected	-	-	-	-	-
8.98	(+)-Fargesin	371.1484	370.1406	C_21_H_22_O_6_	137.0597	156.0567	217.0858	285.0750	-	-	Detected	-	-	-
9.00	Eschweilenol C	449.0709	448.0631	C_20_H_16_O_12_	317.0287	285.0024	257.0073	302.0053	-	-	-	-	Detected	-
9.01	Ellagic acid 2-rhamnoside	449.0708	448.0630	C_20_H_16_O_12_	317.0287	285.0024	257.0075	302.0054	-	-	-	Detected	-	-
9.03	(−)-8-Prenylnaringenin	341.1377	340.1299	C_20_H_20_O_5_	137.0595	248.0821	203.0850	291.1010	-	-	Detected		Detected	-
9.03	Licoflavanone	341.138	340.1302	C_20_H_20_O_5_	137.0597	114.0913	203.0850	227.0699	-	-		Detected	-	-
9.08	Edpetiline	592.3893	591.3815	C_33_H_53_NO_8_	89.0599	133.0858	575.3634	99.0807	-	-	Detected	-	-	-
9.13	Oleuropeinol	363.1644	362.1566	C_16_H_26_O_9_	69,0340	87.0444	155.0703		Detected	-	-	-	-	-
9.16	3-O-Methylellagic acid	317.0287	316.0209	C_15_H_8_O_8_	257.0088	285.0027	201.0181	145.0282	-	-	-	-	Detected	-
9.18	Kaempferol-3-Glucuronide	463.0866	462.0788	C_21_H_18_O_12_	317.0287	285.0026	257.0075	302.0057	-	-	-	-	Detected	-
9.30	Morachalcone A	341.1378	340.1300	C_20_H_20_O_5_	137.0596	248.0836	291.1011	211.0755	-	-	Detected	-	Detected	-
9.36	5-(2-Oxopropyl) hygrine	198.1486	197.1408	C_11_H_19_NO_2_	55.0548	91.0544	69.0704	79.0547	-	-	-	Detected	-	-
9.52	Pinobanksin	273.0753	272.0675	C_15_H_12_O_5_	153.0179	91.0545	119.0491		-	-	Detected	-	-	-
9.53	Dihydrogenistein	273.0753	272.0675	C_15_H_12_O_5_	153.0179	91.0545	119.0490	147.0437	-	-	-	Detected	-	-
9.82	Angustin B	333.0964	332.0886	C_17_H_16_O_7_	137.0596	167.0336	163.0387	122.0362	-	-	-	-	Detected	-
9.83	Blumeatin B	333.0964	332.0886	C_17_H_16_O_7_	137.0597	167.0336	122.0365	259.0963	-	-	-	-	-	Detected
9.90	Elaeokanine C	212.1641	211.1563	C_12_H_21_NO_2_	55.0549	69.0704	79.0547	91.0546	Detected	-	-	-	Detected	Detected
10.12	Coumarandione	149.023	148.0152	C_8_H_4_O_3_	65.0392	53.0028	88.7265	111.4721	Detected	Detected	Detected	Detected	Detected	Detected
10.24	7-Methoxycoumarin	177.0544	176.0466	C_10_H_8_O_3_	65.0391	121.0286	149.0233	91.0546	Detected	Detected	Detected	Detected	Detected	Detected
10.34	Dichotomocej A	226.1796	225.1718	C_13_H_23_NO_2_	55.0549	79.0547	67.0547	180.1744	Detected	Detected	Detected	Detected	-	Detected
10.53	Antiarol	185.0805	184.0727	C_9_H_12_O_4_	68.9976	129.0179	87.0078		Detected	-	-	-	-	
10.54	Umbellatolide B	185.0807	184.0729	C_9_H_12_O_4_	185.0807	184.0729	185.0807	184.0729	-	-	-	-	-	Detected
10.55	Urechitol B	305.1588	304.1510	C_14_H_24_O_7_	129.0182	68.9976	185.0807	139.0023	Detected	-	-	-	-	-
10.63	4-Heptylresorcinol	209.1533	208.1455	C_13_H_20_O_2_	91,0545	105.0702	79.0546	179.1066	Detected	-	Detected	Detected	Detected	-
10.65	5-Heptylresorcinol	209.1533	208.1455	C_13_H_20_O_2_	91.0546	55.0548	105.0700	179.1062	-	Detected	-	-	-	-
10.79	Embelin	295.1900	294.1822	C_17_H_26_O_4_	57.0705	73.0289	101.0236	221.1165	Detected	Detected	-	Detected	Detected	Detected
10.80	(+)-[6]-Gingerol	295.1899	294.1821	C_17_H_26_O_4_	57.0705	73.0289	221.1169	101.0236	-	-	Detected	-	-	-
11.09	Farnesoic acid	237.1845	236.1767	C_15_H_24_O_2_	57.0705	121.1011	181.1219	79.0547	Detected	-	-	-	-	-
11.18	5-O-Methyl embelin	309.2055	308.1977	C_18_H_28_O_4_	57.0705	221.1164	107.0493	165.0546	-	Detected	-	-	-	Detected
11.35	Valerenic acid	235.1689	234.1611	C_15_H_22_O_2_	57.0705	179.1062	107.0494	123.0444	Detected	-	-	Detected	Detected	Detected
11.35	Drimenin	235.1690	234.1612	C_15_H_22_O_2_	57.0705	179.1060	91.0547	107.0491	-	-	Detected	-	-	-
11.42	Kulactone	453.3355	452.3277	C_30_H_44_O_3_	119.0855	107.0857	145.1010	95.0858	-	-	Detected	-	-	-
11.44	Lucialdehyde B	453.3354	452.3276	C_30_H_44_O_3_	119.0855	107.0856	95.0858	133.1012	-	-	-	Detected	-	Detected
11.44	Ganoderic acid S	453.3355	452.3277	C_30_H_44_O_3_	119.0855	145.1010	189.1632	201.1636	-	-	-	-	Detected	-
11.86	Benzaldehyde	107.0492	106.0414	C_7_H_6_O	51.0236	95.0494	77.0392	105.0451	Detected	-	Detected	Detected	Detected	Detected
12.54	2,5-Dimethyl-3,6-bis(tetradecylamino)-1,4-benzoquinone	559.5188	558.5110	C_36_H_66_N_2_O_2_	280.2632	81.0703	95.0858	263.2365	-	-	-	Detected	-	Detected
12.54	Farnesylacetone	263.2364	262.2286	C_18_H_30_O	67.0547	81.0703	95.0858	105.0701	-	-	Detected	Detected	Detected	-

**Table 2:** Antioxidant activities of acetonic and ethanolic extracts of leaves, barks, and roots of *Cochlospermum angolense* Welw ex Oliv. The results are expressed in terms of maximum inhibition effect (Emax, %) and IC_50_ (μg/mL) values. Emax data are presented as mean ± SD for *n* = 3. * *p* < 0.05 vs. Quercetin and ^†^ *p* < 0.05 vs. Curcumin.

	DPPH^•^		NO		O_2_^•−^		Lipid Peroxidation	
Sample	Emax (%)	IC_50_ (μg/mL)	Emax (%)	IC_50_ (μg/mL)	Emax (%)	IC_50_ (μg/mL)	Emax (%)	IC_50_ (μg/mL)
Quercetin	77.9 ± 4.4	8.6 ± 0.5	74.8 ± 3.5	14.3 ± 0.8	53.1 ± 0.8	102.1 ± 5.2	-	-
Curcumin	108.7 ± 22.1	16.6 ± 1.2	78.2 ± 3.2	55.0 ± 0.3	-	-	97.2 ± 0.3	1.5 ± 0.1
L.E.A	77.8 ± 2.9	12.2 ± 0.7 *	34.8 ± 2.1 *^, †^	250.8 ± 12.5 *^, †^	45.9 ± 2.7 ^†^	Nd	93.9 ± 0.8	2.5 ± 0.2 *
L.E.E	83.8 ± 0.2	11.8 ± 0.6 *	63.3 ± 1.7	44.4 ± 2.3 *	38.9 ± 1.6	Nd	92.8 ± 0.7	8.4 ± 0.5 *
B.E.A	77.8 ± 1.1	2.6 ± 0.2 *^, †^	65.9 ± 2.1	24.7 ± 1.3 *^, †^	54.6 ± 3.4	96.4 ± 4.9	95.5 ± 0.1	3.8 ± 0.3 *
B.E.E	78.3 ± 0.9	2.9 ± 0.2 *^, †^	42.9 ± 1.7 *	Nd	55.8 ± 0.5	Nd	93.9 ± 0.5	4.9 ± 0.3 *
R.E.A	12.7 ± 3.2	Nd	55.5 ± 7.3	Nd	37.3 ± 1.3 ^†^	Nd	94.5 ± 0.5	5.9 ± 0.4 *
R.E.E	53.0 ± 6.9 ^†^	16.4 ± 0.9 *	53.5 ± 0.5	101.1 ± 5.1 *	41.0 ± 3.7 ^†^	Nd	66.6 ± 19.2 *	84.4 ± 4.3 *

L.E.A: Leaves acetone extract; L.E.E: Leaves ethanolic extract; B.E.A: Barks acetone extract; B.E.E: Barks ethanolic extract; R.E.A: Roots acetone extract; R.E.E: Roots ethanolic extract; Nd: not determined.

**Table 3:** The different masses (g) and yields (%) of the acetone and ethanolic extracts from the leaves, bark, and roots.

Plant Material	Initial Mass	H.E g	A.E g	E.E g	% H.E	% A.E	% E.E
Leaves	308.97	8.88	9.15	9.48	2.8	3.0	3.2
Barks	544.31	6.30	7.65	7.69	1.1	1.4	1.4
Roots	720.82	4.87	6.96	5.12	0.6	0.9	0.7

A.E. Acetone extract; E.E. Ethanolic extract; H.E. Hexane extract.

## Data Availability

The original contributions presented in this study are included in the article. Further inquiries can be directed to the corresponding authors.

## Supplementary Materials

The following supporting information can be downloaded at: https://www.mdpi.com/article/10.3390/molecules30132768/s1.
