# Supplementary material for: Cochlospermum angolense Welw ex Oliv: Phytochemical Profile, Antioxidant Activity, and Therapeutic Prospects
Source: Molecules. 2025 Jun 27;30(13):2768. doi: 10.3390/molecules30132768 (PMC12251057; doi:10.3390/molecules30132768)
Supplement: Supplementary file 1 [file molecules-30-02768-s001.zip › molecules-3661214-supplementary.pdf]

Supplementary File: Results of HPLC-ESI-MS-MS of *Cochlospermum angolense* Welw ex Oliv leaves, barks and roots ac-etonic and ethanolic extracts.

Table S1. Identification of phytochemicals compounds found in the acetonic extract of *Cochlospermum angolense* leaves by HPLC-ESI-MSn.

Nº	RT	$[M+H]^+$	MW Calc	Formula	Compounds	Class of compounds
1	1.28	102.1280	101.1201	$C_6H_{115}N$	Hexylamine	Amine
2	5.04	125.0389	124.0311	$C_5H_4N_2O_2$	4-Pyrimidinecarboxylic acid	Carboxylic acid
3	5.31	227.1750	226.1672	$C_{12}H_{22}N_2O_2$	Crotetamide	Amide
4	5.33	239.1486	238.1408	$C_{10}H_{22}O_6$	3,3'-[1,2-Ethanediylbis(oxy)]bis(2-methyl-1,2-propanediol)	Alcohol
5	5.37	195.1225	194.1147	$C_8H_{18}O_5$	Peg-4	Alcohol
6	6.16	300.2013	299.1935	$C_{13}H_{25}N_5O_3$	L-Prolyl-L-lysylglycinamide	Peptide
7	6.46	283.1748	282.1669	$C_{12}H_{26}O_7$	3,6,9,12,15-Pentaoxaheptadecan-1,17-diol	Alcohol
8	6.93	327.2008	326.1930	$C_{14}H_{30}O_8$	Heptaethylene glycol	Alcohol
9	7.08	344.2274	343.2196	$C_{15}H_{29}N_5O_4$	2-[-2-aminopropanamido]-N-[(1S)-1-[(2,2-dimethylpropyl)carbamoyl]ethyl]butanediamide	Amide
10	7.34	324.2276	323.2198	$C_{17}H_{29}N_3O_3$	Stravidin	Protein
11	7.36	388.2534	387.2456	$C_{17}H_{33}N_5O_5$	Glutaminyl-leucyl-lysine	Protein
12	7.60	432.2796	431.2717	$C_{19}H_{37}N_5O_6$	Istamycin C1	Formamide
13	7.65	217.1069	216.0990	$C_{10}H_{16}O_5$	Diethyl β-ketoadipate	Ester
14	7.73	311.2060	310.1982	$C_{14}H_{30}O_7$	3,6,9,12,15,18-Hexaoxaicosan-1-ol	Alcohol
15	7.75	476.3060	475.2982	$C_{21}H_{41}N_5O_7$	Netilmicin	Aminoglycoside
16	7.81	198.1275	197.1197	$C_{14}H_{15}N$	Dibenzylamine	Amine
17	7.93	520.3323	519.3245	$C_{23}H_{45}N_5O_8$	2-[4-amino-3-[3-amino-6-[(2,3-dihydroxypropylamino)methyl]-3,4-dihydro-2H-pyran-2-yl]-2-hydroxy-6-(methylamino)cyclohexyl]oxy-5-methyl-4-(methylamino)oxane-3,5-diol	Glycoside
18	7.98	306.2460	305.2382	$C_{19}H_{31}NO_2$	Samandarin	Alcaloide
19	7.99	469.3378	468.3302	$C_{23}H_{48}O_9$	5,8,11,14,17,21,24,27-Octaoxahentriacontan-19-ol	Alcohol
20	8.03	113.0599	112.0521	$C_6H_8O_2$	Sorbic acid	Fatty acid
21	8.04	219.1226	218.1147	$C_{10}H_{18}O_5$	3-Hydroxysebacic acid	Carboxylic acid
22	8.05	355.2322	354.2243	$C_{16}H_{34}O_8$	3,6,9,12,15,18,21-Heptaoxatricosan-1-ol	Alcohol
23	8.08	564.3583	563.3505	$C_{25}H_{49}N_5O_9$	3-amino-N'-[(1R,5S)-5-amino-4-[(2R,6R)-3-amino-6-(4-hydroxybutyl)oxan-2-yl]oxy-2-[(2R,4R)-3,5-dihydroxy-4,5-dimethyloxan-2-yl]oxy-3-hydroxycyclohexyl]-2-hydroxypropanimidamide	Amide
24	8.14	453.3427	452.3348	$C_{23}H_{48}O_8$	2,2,6,6,10,10,14,14-Octamethyl-1,3,5,7,9,11,13,15-pentadecaneoctol	Alcohol
25	8.22	399.2583	398.2505	$C_{18}H_{38}O_9$	3,6,9,12,15,18,21,24-Octaoxahexacosan-1-ol	Alcohol
26	8.26	311.2060	310.1981	$C_{14}H_{30}O_7$	3,6,9,12,15,18-Hexaoxaicosan-1-ol	Alcohol
27	8.42	453.3427	452.3350	$C_{23}H_{48}O_8$	2,2,6,6,10,10,14,14-Octamethyl-1,3,5,7,9,11,13,15-pentadecaneoctol	Alcohol

28	8.45	163.1327	162.1249	$C_8H_{18}O_3$	1,2,3-octanetriol	Alcohol
29	8.47	183.1014	182.0936	$C_{10}H_{14}O_3$	Hydroconiferyl alcohol	Flavonoide
30	8.55	566.4267	565.4192	$C_{30}H_{55}N_5O_5$	Clavatustide C	Peptide
31	8.67	460.3110	459.3032	$C_{21}H_{41}N_5O_6$	H-leu-ser-lys-leu-oh	Peptide
32	8.73	241.2033	240.1955	$C_{12}H_{24}N_4O$	2-{3-[2-(Diethylamino)ethyl]-1,2,4-oxadiazol-5-yl}-N-methyl-2-propanamine	Amine
33	8.78	228.1492	227.1415	$C_{14}H_{17}N_3$	3-(Aminomethyl)-N-benzyl-N-methyl-2-pyridinamine	Amine
34	8.79	504.3371	503.3293	$C_{23}H_{45}N_5O_7$	6'-N-tert-Butylsisomicin	Amineglycoside
35	8.80	443.2844	442.2766	$C_{20}H_{42}O_{10}$	2,4,6,8,10,12,14,16,18-Nonaoxanonacosan-1-ol	Alcohol
36	8.85	487.3106	486.3028	$C_{22}H_{46}O_{11}$	Etoxipoli(etilenglicol) 10	Alcohol
37	8.89	340.2591	339.2513	$C_{18}H_{33}N_3O_3$	1-(2-methylbutanoyl)-N-[3-(morpholin-4-yl)propyl]piperidine-4-carboxamide	Amide
38	8.97	207.1589	206.1511	$C_{10}H_{22}O_4$	1-[2-(2-methoxy-1-methylethoxy)-1-methylethoxy]-2-Propanol	Alcohol
39	8.98	133.0648	132.0570	C_9H_8O	Cinnamaldehyde	Aldehyde
40	9.03	548.3635	547.3557	$C_{26}H_{45}N_9O_4$	NI	
41	9.04	592.3898	591.3820	$C_{39}H_{49}N_3O_2$	Carbamic acid, [1,1'-biphenyl]-2-yl-, 4-methyl-1-[8-[methyl(1-naphthalenylmethyl)amino]octyl]-4-piperidinyl ester	Ether
42	9.13	363.1644	362.1566	$C_{16}H_{26}O_9$	Oleuropeinol	Phenol acid
43	9.28	371.2509	370.2431	$C_{19}H_{34}N_2O_5$	Boc-Gly(cpent)-Gly(tbu)-Ome	Peptide
44	9.30	636.4158	635.4080	$C_{41}H_{53}N_3O_3$	14-(1-benzylpyrazol-4-yl)-11-cyano-2,2,6a,6b,9,9,12a-heptamethyl-10-oxo-3,4,5,6,6a,7,8,8a,11,12,13,14b-dodecahydro-1H-picene-4a-carboxylic acid	Carboxylic acid
45	9.33	309.2267	308.2189	$C_{15}H_{32}O_6$	1-o-nonylhexitol	Alcohol
46	9.35	198.1486	197.1408	$C_{11}H_{19}NO_2$	1-(2-Ethyl-1-piperidinyl)-1,3-butanedione	Ketone
47	9.40	200.1643	199.1565	$C_{11}H_{21}NO_2$	N,N-Dimethyl-3-oxononanamide	Amide
48	9.43	219.0470	218.0392	$C_8H_{10}O_7$	(2R,4R,4as,8ar)-2-cyclohexyloctahydro-2H-chromen-4-ol	Alcohol
49	9.57	415.2769	414.2691	$C_{28}H_{34}N_2O$	1-[2-(Diphenylmethoxy)ethyl]-4-(3-phenylpropyl)-piperazin	Aromatic hydrocarbon
50	9.61	437.2903	436.2825	$C_{25}H_{40}O_6$	Tenivastatin	Hydroxylic acid
51	9.66	459.3032	458.2954	$C_{19}H_{38}N_8O_5$	H-lys-arg-gly-val-oh	Protein
52	9.70	214.0893	213.0815	$C_{13}H_{11}NO_2$	Salicylanilide	Amide
53	9.71	481.3163	480.3085	$C_{27}H_{44}O_7$	20-hydroxyecdysterone	Sterol
54	9.73	205.1432	204.1354	$C_{10}H_{20}O_4$	3,5-Dihydroxydecanoic acid	Fatty acid
55	9.77	249.1846	248.1758	$C_{16}H_{24}O_2$	Methyl 4-octylbenzoate	Carboxylic acid ether
56	9.90	212.1641	211.1563	$C_{12}H_{21}NO_2$	Elaeokanine C	Alcaloide
57	9.92	552.2644	551.2565	$C_{24}H_{41}NO_{13}$	NI	-

58	9.94	187.1326	186.1248	$C_{10}H_{18}O_3$	Methyl 9-oxononanoate	Carboxylic acid ether
59	10.12	149.0230	148.0152	$C_8H_4O_3$	Coumarandione	Coumarin
60	10.17	638.3008	637.2930	$C_{41}H_{39}N_3O_4$	9-Butyl-9H-carbazole-3-yl 2-(p-acetylphenyl)-4,6-dihydroxy-4,6-bis(2-pyridyl)cyclohexyl ketone	Ketone
61	10.23	158.1538	157.1460	$C_9H_{19}NO$	Nonanamide	Amide
62	10.24	177.0544	176.0466	$C_{10}H_8O_3$	Herniarin	Coumarin
63	10.29	279.0927	278.0851	$C_8H_{14}N_4O_7$	Diazolidinyl urea	Amide
64	10.34	226.1796	225.1718	$C_{13}H_{23}NO_2$	Dichotomocej A	Diterpene
65	10.40	228.1951	227.1873	$C_{13}H_{25}NO_2$	Decyloxazolidinone	Ketone
66	10.43	724.3372	723.3294	$C_{45}H_{45}N_3O_6$	2,2',2''-[(2,4,6-Tributylbenzene-1,3,5-triyl)tris(methylene)]tris(1H-isoindole-1,3(2H)-dione)	Ketone
67	10.51	240.1953	239.1875	$C_{14}H_{25}NO_2$	N,N-Dimethyl-4-oxo-2-dodecenamide	Amide
68	10.52	259.1536	258.1458	$C_{13}H_{22}O_5$	Diethyl 3-oxononanedioate	Ether
69	10.53	185.0805	184.0727	$C_9H_{12}O_4$	Antiarol	Phenol
70	10.54	203.0910	202.0832	$C_9H_{14}O_5$	(4-Ethyl-3-hydroxy-5-oxooxolan-3-yl)methyl acetate	Ether
71	10.55	305.1588	304.1510	$C_{14}H_{24}O_7$	Urechitol B	Terpenoid
72	10.61	183.0803	182.0725	$C_{13}H_{10}O$	9-fluorenol	Alcohol
73	10.63	209.1533	208.1455	$C_{13}H_{20}O_2$	4-heptylresorcinol	Phenol
74	10.68	282.2786	281.2708	$C_{18}H_{35}NO$	(9z)-9-octadecenamide	Amide
75	10.76	250.1181	249.1103	$C_{17}H_{15}NO$	1-Benzyl-4-methyl-2(1H)-quinolinone	Ketone
76	10.79	295.1900	294.1822	$C_{17}H_{26}O_4$	Embelin	Phenol
77	10.80	161.0959	160.0881	$C_{11}H_{12}O$	4-(p-Tolyl)-3-buten-2-one	Ketone
78	10.85	141.0908	140.0830	$C_8H_{12}O_2$	Oct-2-ynoic acid	Carboxylic acid
79	10.95	221.1169	220.1091	$C_{13}H_{16}O_3$	(3Z)-3-Hexen-1-yl salicylate	Ether
80	11.01	172.1693	171.1615	$C_{10}H_{21}NO$	Decanamide	Amide
81	11.09	237.1845	236.1767	$C_{15}H_{24}O_2$	Farnesoic acid	Terpenoid
82	11.18	309.2788	308.1978	$C_{18}H_{28}O_4$	Ostopanic acid	Fatty acid
83	11.35	235.1689	234.1611	$C_{15}H_{22}O_2$	Valerenic acid	Terpenoid
84	11.66	149.0230	148.0152	$C_8H_4O_3$	Phthalic anhydride	Anhydride
85	11.67	259.1534	258.1456	$C_{13}H_{22}O_5$	6-Oxo-undecanedioic acid, dimethyl ester	Ether
86	11.68	361.2212	360.2134	$C_{18}H_{32}O_7$	Methyl 2,4-di-O-acetyl-3-O-octylpentopyranoside	Carbohydrate
87	11.74	200.2003	199.1925	$C_{12}H_{25}NO$	Lauramide	Amide
88	11.84	310.2369	309.2292	$C_{18}H_{31}NO_3$	A-Heptyl-3,4,5-trimethoxyphenethylamine	Amide
89	11.86	107.0492	106.0414	C_7H_6O	Benzaldehyde	Aldehyde
90	11.96	403.2318	402.2240	$C_{20}H_{34}O_8$	Botcinolide	Lactone

91	12.26	228.2317	227.2239	$C_{14}H_{29}NO$	Myristamide	Amide
92	12.55	263.2363	262.2285	$C_{18}H_{30}O$	Farnesylacetone	Ketone
93	12.57	280.2628	279.2550	$C_{18}H_{33}NO$	Linoleamide	Amide
94	12.59	559.5189	558.5111	$C_{36}H_{66}N_2O_2$	2,5-Dimethyl-3,6-bis(tetradecylamino)-1,4-benzoquinone	Quinone
95	12.75	256.2628	255.2550	$C_{16}H_{33}NO$	Palmitamide	Amide
96	12.88	304.2605	303.2527	$C_{20}H_{33}NO$	Arachidonamide	Amide
97	12.89	563.5501	562.5423	$C_{36}H_{70}N_2O_2$	1,1′-Dihydropyrimidine-1,3(2H,4H)-diyldi(1-hexadecanone)	Ketone
98	13.42	310.3098	309.3020	$C_{20}H_{39}NO$	Cis-11-Eicosenamide	Amide
99	14.14	338.3409	337.3331	$C_{22}H_{43}NO$	Erucamide	Amide
100	17.19	663.4527	662.4446	$C_{44}H_{58}N_2O_3$	3-(4-Decoxyphenyl)-5-[4-[2-(4-dodecoxyphenyl)ethynyl]phenyl]-1,2,4-oxadiazole	Oxadiazols

RT = retention time; $[M+H]^+$ = measured mass as positive ion (m/z); MW = molecular mass; NI: unidentified.

Table S2. Identification of phytochemicals compounds found in the ethanolic extract of *Cochlospermum angolense* leaves by HPLC-ESI-MSn.

Nº	RT	$[M+H]^+$	MW Calc	Formula	Compounds	Class of compounds
1	0.04	282.2788	281.2710	$C_{18}H_{35}NO$	(9z)-9-octadecenamide	Amide
2	1.05	162.0760	161.0682	$C_6H_{11}NO_4$	A-Aminoadipic acid	Amino acid
3	1.10	104.1072	103.0994	$C_5H_{13}NO$	L-valinol	Amino alcohol
4	1.16	118.0863	117.0785	$C_5H_{11}NO_2$	Valine	Amino acid
5	1.17	175.0574	174.0496	$C_7H_{10}O_5$	(−)-Shikimic acid	Carboxylic acid
6	1.24	102.1280	101.1202	$C_6H_{15}N$	Hexylamine	Amine
7	1.26	116.1070	115.0993	$C_6H_{13}NO$	N-Hexanamide	Amide
8	1.93	130.0499	129.0421	$C_5H_7NO_3$	Pyroglutamic acid	Amino acid
9	5.19	125.0389	124.0311	$C_5H_4N_2O_2$	Pyrazinoic acid	Carboxylic acid
10	6.37	239.1486	238.1408	$C_{10}H_{22}O_6$	2,3,4,6-Tetra-O-methyl-D-galactitol	Alcohol
11	6.42	227.1752	226.1673	$C_{12}H_{22}N_2O_2$	Crotetamide	Amide
12	6.84	563.5503	562.5425	$C_{36}H_{70}N_2O_2$	1,1'-[(E)-Diazenediyl]di(octadecan-1-one)	Ketone
13	6.87	300.2014	299.1936	$C_{13}H_{25}N_5O_3$	L-Prolyl-L-lysylglycinamide	Peptide
14	7.10	327.2010	326.1932	$C_{14}H_{30}O_8$	Tetradecane-1,5-α,6-α,7-α,8-α,9-α,10-β,11-α-octaol	Alcohol
15	7.12	344.2274	343.2196	$C_{15}H_{29}N_5O_4$	(2S)-2-[(2S)-2-aminopropanamido]-N-[(1S)-1-[(2,2-dimethylpropyl)carbamoyl]ethyl]butanediamide	Amide
16	7.35	388.2534	387.2456	$C_{17}H_{33}N_5O_5$	Gln-leu-lys	Peptide
17	7.41	420.1859	419.1781	$C_{19}H_{25}N_5O_6$	Ser-trp-gln	Peptide
18	7.47	340.2589	339.2511	$C_{18}H_{33}N_3O_3$	1-Acetamido-N-{[1-(2-methoxyethyl)-4-piperidinyl]methyl}cyclohexanecarboxamide	Amide
19	7.58	432.2798	431.2720	$C_{19}H_{37}N_5O_6$	H-DL-xiile-DL-xithr-DL-Lys-DL-Ala-OH	Peptide
20	7.84	198.1276	197.1197	$C_9H_{15}N_3O_2$	Hercynine	Amino acid
21	7.86	217.1069	216.0991	$C_{10}H_{16}O_5$	Ethyl acetylsuccinate	Ether
22	7.94	149.0121	148.0043	$C_4H_4O_6$	Dihydroxyfumaric acid	Carboxylic acid
23	7.97	449.1073	448.0995	$C_{21}H_{20}O_{11}$	Quercitrin	Flavonoid
24	8.10	449.1072	448.0994	$C_{21}H_{20}O_{11}$	Astragalin	Flavonoid
25	8.14	453.3429	452.3351	$C_{23}H_{48}O_8$	(3S,5S,7R,9R,11S,13S)-2,2,6,6,10,10,14,14-Octamethyl-1,3,5,7,9,11,13,15-pentadecaneoctol	Alcohol
26	8.20	433.1125	432.1047	$C_{21}H_{20}O_{10}$	Vitexin	Flavonoid
27	8.38	433.1122	432.1044	$C_{21}H_{20}O_{10}$	Apigetrin	Flavonoid
28	8.55	479.0813	478.0735	$C_{21}H_{18}O_{13}$	Quercetin-3'-glucuronide	Flavonoid
29	8.56	566.4269	565.4191	$C_{30}H_{55}N_5O_5$	Viscumamide	Amide
30	8.58	303.0493	302.0415	$C_{15}H_{10}O_7$	Quercetin	Flavonoid

31	8.60	465.1023	464.0945	$C_{21}H_{20}O_{12}$	Isoquercetin	Flavonoid
32	8.70	303.0495	302.0417	$C_{15}H_{10}O_7$	Morin	Flavonoid
33	8.73	435.0916	434.0838	$C_{20}H_{18}O_{11}$	Quercetin-3-arabinoside	Flavonoid
34	8.82	163.1327	162.1249	$C_8H_{18}O_3$	1,2,3-octanetriol	Alcohol
35	8.86	185.1146	184.1068	$C_{10}H_{16}O_3$	9-Oxodecenoic acid	Carboxylic acid
36	8.95	287.0545	286.0467	$C_{15}H_{10}O_6$	Kaempferol	Flavonoid
37	9.45	219.0470	218.0392	$C_8H_{10}O_7$	6-O-Acetylascorbic acid	Carboxylic acid
38	9.74	214.0894	213.0816	$C_{13}H_{11}NO_2$	8-Quinolinyl (2E)-2-butenoate	Ether
39	9.87	212.0648	211.1565	$C_{12}H_{21}NO_2$	Elaeokanine C	Alkaloid
40	9.91	552.2644	551.2566	$C_{25}H_{37}N_5O_9$	L-Leucine, N-[N-[N-[N-[N-[(phenylmethoxy)carbonyl]glycyl]glycyl]glycyl]-L-seryl]- ethyl ester	Ether
41	9.96	144.1382	143.1304	$C_8H_{17}NO$	N-Octanamide	Amide
42	10.09	149.0231	148.0152	$C_8H_4O_3$	Coumarandione	Phenol (coumarin)
43	10.11	177.0544	176.0466	$C_{10}H_8O_3$	7-methoxycoumarin (herniarin)	Coumarin (lactone)
44	10.18	638.3008	637.2930	$C_{41}H_{39}N_3O_4$	1-[4-[2-(9-Butylcarbazole-3-carbonyl)-3,5-dihydroxy-3,5-dipyridin-2-ylcyclohexyl]phenyl]ethanone	Ketone
45	10.26	158.1538	157.1460	$C_9H_{19}NO$	Nonanamide	Amide
46	10.28	279.0928	278.0850	$C_{13}H_{14}N_2O_5$	Pukeleimide A	Amide
47	10.30	226.1798	225.1720	$C_{13}H_{23}NO_2$	Dichotomocej A	Terpene
48	10.40	228.1953	227.1875	$C_{13}H_{25}NO_2$	Cyprodenate	Benzodiazepine
49	10.52	305.1590	304.1512	$C_{14}H_{24}O_7$	(6R)-6-[(1R)-2-Acetoxy-1-hydroxyethyl]-1-O-acetyl-3,6-anhydro-4,5-dideoxy-6-methyl-2-C-methyl-D-erythro-hexitol	Alcohol
50	10.65	209.1533	208.1455	$C_{13}H_{20}O_2$	5-heptylresorcinol	Phenol
51	10.80	295.1900	294.1822	$C_{17}H_{26}O_4$	Embelin	Phenol
52	11.01	172.1693	171.1615	$C_{10}H_{21}NO$	Decanamide	Amide
53	11.21	309.2054	308.1977	$C_{18}H_{28}O_4$	5-O-Methyl embelin (monohydroxi-1,4-benzoquinone)	Quinone
54	11.47	200.2005	199.1927	$C_{12}H_{25}NO$	Lauramine	Amine
55	11.66	361.2214	360.2113	$C_{18}H_{32}O_7$	Methyl 2,4-di-O-acetyl-3-O-octylpentopyranoside	Glycoside
56	11.69	259.1535	258.1457	$C_{13}H_{22}O_5$	6-Oxoundecanedioic acid dimethyl ester	Ether
57	11.76	200.2004	199.1926	$C_{12}H_{25}NO$	N,n-dimethyldecanamide	Amide
58	11.85	310.2370	309.2292	$C_{18}H_{31}NO_3$	Melophlin M	Pyrrolidine
59	12.27	228.2316	227.2237	$C_{14}H_{29}NO$	Myristamide	Amide
60	12.57	280.2628	279.2550	$C_{18}H_{33}NO$	Linoleamide	Amide
61	12.78	256.2627	255.2549	$C_{16}H_{33}NO$	Palmitamide	Amide

62	12.85	304.2605	303.2526	$C_{20}H_{33}NO$	Arachidonamide	Amide
63	12.88	563.5500	562.5422	$C_{36}H_{70}N_2O_2$	1-(3-Hexadecanoyl-4-methylimidazolidin-1-yl)hexadecan-1-one	Ketone
64	14.14	338.3410	337.3332	$C_{22}H_{43}NO$	Erucamide	Amide

RT = retention time; $[M+H]^+$ = measured mass as positive ion (m/z); MW = molecular mass; NI: unidentified.

Table S3. Identification of phytochemicals compounds found in the acetonic extract of *Cochlospermum angolense* barks by HPLC-ESI-MSn.

Nº	RT	[M+H]+	MW Calc	Formula	Compounds	Class of compounds
1	0.03	282.2787	281.2709	$C_{18}H_{35}NO$	(9Z)-9-Octadecenamide	Amide
2	0.12	343.3387	342.3309	$C_{25}H_{42}$	1-Hexadecylindane	Aromatic hydrocarbon
3	1.21	102.1280	101.1202	$C_6H_{15}N$	Hexylamine	Amine
4	4.91	125.0389	124.0311	$C_5H_4N_2O_2$	Pyrazinoic acid	Carboxylic acid
5	5.93	195.1226	194.1148	$C_8H_{18}O_5$	2,4-Di-O-methylfucitol	Alcohol
6	5.96	149.0120	148.0042	$C_4H_4O_6$	Dihydroxymaleic acid	Carboxylic acid
7	6.10	227.1751	226.1673	$C_{12}H_{22}N_2O_2$	Crotetamide	Amide
8	6.22	239.1486	238.1408	$C_{10}H_{22}O_6$	Tetra-O-methylsorbitol	Alcohol
9	6.71	130.1589	129.1511	$C_8H_{19}N$	Octylamine	Amine
10	6.76	300.2011	299.1934	$C_{13}H_{25}N_5O_3$	L-Prolyl-L-lysylglycinamide	Amide
11	7.03	563.5502	562.5424	$C_{36}H_{70}N_2O_2$	1,1'-[(E)-Diazenediyl]di(octadecan-1-one)	Ketone
12	7.11	327.2796	326.1930	$C_{14}H_{30}O_8$	Tetradecane-1,5-α,6-α,7-α,8-α,9-α,10-β,11-α-octaol	Alcohol
13	7.13	344.2272	343.2196	$C_{15}H_{29}N_5O_4$	(2S)-2-[(2S)-2-Aminopropanamido]-N-[(1S)-1-[(2,2-dimethylpropyl)carbamoyl]ethyl]butanediamide	Amide
14	7.29	324.2278	323.2200	$C_{17}H_{29}N_3O_3$	Stravidin	Protein
15	7.36	388.2534	387.2456	$C_{17}H_{33}N_5O_5$	Gln-Leu-Lys	Peptide
16	7.37	371.2271	370.2193	$C_{16}H_{34}O_9$	HO-dPEG8-OH	Alcohol
17	7.59	432.2798	431.2720	$C_{19}H_{37}N_5O_6$	Istamycin C1	Amide
18	7.62	340.2589	339.2511	$C_{18}H_{33}N_3O_3$	1-Acetamido-N-{[1-(2-methoxyethyl)-4-piperidinyl]methyl}cyclohexanecarboxamide	Amide
19	7.76	476.3060	475.2982	$C_{21}H_{41}N_5O_7$	Netilmicin	Aminoglycoside
20	7.81	311.2060	310.1982	$C_{14}H_{30}O_7$	3,6,9,12,15,18-Hexaoxaicosan-1-ol	Alcohol
21	7.83	198.1275	197.1197	$C_9H_{15}N_3O_2$	Hercynine	Amino acid
22	7.87	217.1068	216.0990	$C_{10}H_{16}O_5$	3-Oxo-1,8-octanedicarboxylic acid	Carboxylic acid
23	7.96	520.3321	519.3243	$C_{23}H_{45}N_5O_8$	2-[4-amino-3-[3-amino-6-[(2,3-dihydroxypropylamino)methyl]-3,4-dihydro-2H-pyran-2-yl]-2-hydroxy-6-(methylamino)cyclohexyl]oxy-5-methyl-4-(methylamino)oxane-3,5-diol	Alcohol
24	8.07	355.2322	354.2244	$C_{17}H_{30}N_4O_4$	Ethyl (3R,4R,5S)-4-acetamido-5-(diaminomethylideneamino)-3-pentan-3-yloxycyclohexene-1-carboxylate	Ether-amide
25	8.11	219.1013	218.0935	$C_{13}H_{14}O_3$	Eupatoriochromene	Chromene
26	8.14	453.3429	452.3351	$C_{23}H_{48}O_8$	(3S,5S,7R,9R,11S,13S)-2,2,6,6,10,10,14,14-Octamethyl-1,3,5,7,9,11,13,15-pentadecaneoctol	Alcohol
27	8.36	355.2320	354.2242	$C_{16}H_{34}O_8$	3,6,9,12,15,18,21-Heptaoxatricosan-1-ol	Alcohol
28	8.47	273.0752	272.0674	$C_{15}H_{12}O_5$	Naringenin	Flavonoid

29	8.57	566.4267	565.4189	$C_{35}H_{55}N_3O_3$	N-[(E,2S,3R)-3-hydroxy-1-[(6-pyridin-2-ylpyridin-2-yl)methoxy]octadec-4-en-2-yl]hexanamide	Amide
30	8.62	289.0703	288.0625	$C_{15}H_{12}O_6$	(+)-Dihydrokaempferol	Flavonoid
31	8.64	219.1377	218.1299	$C_{14}H_{18}O_2$	Cyclohexyl phenylacetate	Ether
32	8.82	163.1327	162.1249	$C_8H_{18}O_3$	Diethylene glycol n-butyl ether	Ether
33	8.84	185.1146	184.1068	$C_{10}H_{18}O_3$	9-Oxodecenoic acid	Carboxylic acid
34	8.97	548.3632	547.3554	$C_{32}H_{45}N_5O_3$	(3S,6S,7R,9aS)-6-{[(2S)-2-Aminobutanoyl]amino}-7-[(diethylamino)methyl]-N-(diphenylmethyl)-5-oxooctahydro-1H-pyrrolo[1,2-a]azepine-3-carboxamide	Amide
35	8.98	371.1484	370.1406	$C_{21}H_{22}O_6$	(+)-Fargesin	lignan
36	9.03	341.1377	340.1299	$C_{20}H_{20}O_5$	(−)-8-Prenylnaringenin	Flavonoid
37	9.08	592.3895	591.3815	$C_{33}H_{53}NO_8$	Edpetiline	Alkaloid
38	9.15	636.4158	635.4080	$C_{41}H_{53}N_3O_3$	NI	
39	9.29	175.0752	174.0674	$C_{11}H_{10}O_2$	Menadiol	Alcohol
40	9.31	341.1378	340.1300	$C_{20}H_{20}O_5$	Morachalcone A	Flavonoid
41	9.38	198.3898	197.1408	$C_{11}H_{19}NO_2$	N-butyl-3-oxocyclohexane-1-carboxamide	Amide
42	9.44	219.0471	218.0393	$C_8H_{10}O_7$	6-O-Acetylascorbic acid	Carboxylic acid
43	9.52	273.0753	272.0675	$C_{15}H_{12}O_5$	Pinobanksin	Flavanol
44	9.74	214.0893	213.0815	$C_{13}H_{11}NO_2$	Salicylanilide	Amide
45	9.83	333.0963	332.0886	$C_{17}H_{16}O_7$	Sulochrin	Methyl ester
46	9.87	212.1642	211.1564	$C_{12}H_{21}NO_3$	N-((8E)-3-Hydroxy-5-oxodec-8-en-1-yl)ethanimidate	Amida
47	9.91	552.2642	551.2564	$C_{25}H_{37}N_5O_9$	N-(benzyloxycarbonyl)glycylglycylglycyl-L-serin-L-leucine ethyl ester	Ester
48	9.97	144.0470	143.1304	$C_8H_{17}NO$	n-Octanamide	Amida
49	10.09	177.0544	176.0466	$C_{10}H_8O_3$	7-Methoxycoumarin (hernianin)	Coumarin (Lactone)
50	10.13	149.0231	148.0153	$C_8H_4O_3$	Coumarandione	Phenol
51	10.17	638.3007	637.2929	$C_{41}H_{39}N_3O_4$	Dibenzyl 4-(3-mesityl-1-phenyl-1H-pyrazol-4-yl)-2,6-dimethyl-1,4-dihydropyridine-3,5-dicarboxylate	Ester
52	10.21	200.1643	199.1565	$C_{11}H_{21}NO_2$	5-(2-Hydroxypropyl)-hygrine	Alkaloid
53	10.26	158.1537	157.1459	$C_9H_{19}NO$	Nonanamide	Amide
54	10.29	279.0929	278.0851	$C_{13}H_{14}N_2O_5$	Pukeleimide A	Amide
55	10.30	226.1798	225.1720	$C_{13}H_{23}NO_2$	Dichotomocej A	Terpenoid
56	10.39	228.1953	227.1875	$C_{13}H_{25}NO_2$	4-Decyloxazolidin-2-one	Ketone
57	10.52	305.1590	304.1512	$C_{14}H_{24}O_7$	(6R)-6-[(1R)-2-Acetoxy-1-hydroxyethyl]-1-O-acetyl-3,6-anhydro-4,5-dideoxy-6-methyl-2-C-methyl-D-erythro-hexitol	Carbohydrate
58	10.54	259.1535	258.1457	$C_{13}H_{22}O_5$	6-Oxoundecanedioic acid dimethyl ester	Ester
59	10.64	209.1532	208.1454	$C_{13}H_{20}O_2$	4-Heptylresorcinol	Phenol

60	10.80	295.1899	294.1821	$C_{17}H_{26}O_4$	(+)-[6]-Gingerol	Phenol
61	10.81	161.0559	160.0881	$C_{11}H_{12}O$	5-Phenylpent-3-en-2-one	Ketone
62	11.02	172.1692	171.1614	$C_{10}H_{21}NO$	Decanamide	Amide
63	11.27	309.2054	308.1975	$C_{18}H_{28}O_4$	Ostopanic Acid	Fatty acid
64	11.29	200.2006	199.1928	$C_{12}H_{25}NO$	Lauramide	Amide
65	11.35	235.1690	234.1612	$C_{15}H_{22}O_2$	Drimenin	Sesquiterpene
66	11.36	291.1950	290.1872	$C_{18}H_{26}O_3$	Ethylhexyl methoxycinnamate	Ester
67	11.42	453.3355	452.3277	$C_{30}H_{44}O_3$	Kulactone	Terpene
68	11.66	361.2214	360.2135	$C_{19}H_{28}N_4O_3$	(2S)-1-[(2S)-2-[(2S)-2-aminopropanamido]-3-methylbutanoyl]-N-phenylpyrrolidine-2-carboxamide	Amide
69	11.84	107.0493	106.0415	C_7H_6O	Benzaldehyde	Aldehyde
70	11.86	310.2370	309.2292	$C_{18}H_{31}NO_3$	Melophlin M	pyrroline
71	11.98	237.1482	236.1404	$C_{14}H_{20}O_3$	Heptyl salicylate	Ester
72	12.26	228.0803	227.2240	$C_{14}H_{29}NO$	Myristamide	Amide
73	12.55	263.2364	262.2286	$C_{18}H_{30}O$	Farnesylacetone	Diterpenoids
74	12.57	280.2627	279.2549	$C_{18}H_{33}NO$	Linoleamide	Fatty acid
75	12.70	561.5345	560.5267	$C_{36}H_{68}N_2O_2$	N-[4-(hexadec-9-enoylamino)butyl]hexadec-9-enamide	Amide
76	12.79	256.2628	255.2550	$C_{16}H_{33}NO$	Palmitamide	Amide
77	12.86	563.5497	562.5419	$C_{36}H_{70}N_2O_2$	1-(3-hexadecanoyl-1,3-diazinan-1-yl)hexadecan-1-one	Ketone
78	12.93	304.2604	303.2526	$C_{20}H_{33}NO$	Arachidonamide	Amide
79	13.43	310.3097	309.3019	$C_{20}H_{39}NO$	cis-11-Eicosenamide	Amide
80	14.20	338.3409	337.3332	$C_{22}H_{43}NO$	Erucamide	Amide

[3] Table: RT = retention time; $[M+H]^+$ = measured mass as positive ion (m/z); MW = molecular mass.

Table S4. Identification of phytochemicals compounds found in the ethanolic extract of *Cochlospermum angolense* barks by HPLC-ESI-MSn.

Nº	RT	$[M+H]^+$	MW Calc	Formula	Compounds	Class of compound
1	0.03	282.2786	281.2708	$C_{18}H_{35}NO$	(9Z)-9-Octadecenamide	Amide
2	1.08	342.1389	341.1311	$C_{12}H_{23}NO_{10}$	Lactosylamine	Aminoglycans (Carbohydrate)
3	1.10	104.1072	103.0994	$C_5H_{13}NO$	5-Aminopentanol	Aminoalcohol
4	1.13	198.0969	197.0892	$C_6H_{15}NO_6$	Aminoglucitol	Aminoglycans (Carbohydrate)
5	1.14	180.0864	179.0786	$C_6H_{13}NO_5$	D-Glucosamine	Aminosaccharide (carbohydrate)
6	1.17	203.0523	202.0445	$C_8H_{10}O_6$	3,5-Dioxooctanedioic acid	Fatty acid
7	1.22	365.1050	364.0972	$C_{14}H_{20}O_{11}$	(3R,4R,5R)-5-[(2R,3R,4S,5S,6R)-6-(formyloxymethyl)-3,4,5-trihydroxyoxan-2-yl]oxy-3,4-dihydroxycyclohexene-1-carboxylic acid	Carboxylic acid
8	1.23	102.1280	101.1202	$C_6H_{15}N$	Hexylamine	Amine
9	1.42	125.0389	124.0311	$C_5H_4N_2O_2$	Pyrazinoic acid	Carboxylic acid
10	5.87	227.1752	226.1674	$C_{12}H_{22}N_2O_2$	Crotetamide	Amide
11	6.11	563.5504	562.5426	$C_{36}H_{70}N_2O_2$	N,N'-ethane-1,2-diylbisoleamide	Amide
12	6.23	239.1487	238.1409	$C_{10}H_{22}O_6$	Tetra-O-methylsorbitol	Carbohydrate
13	6.76	300.2013	299.1935	$C_{13}H_{25}N_5O_3$	L-Prolyl-L-lysylglycinamide	Peptide
14	7.03	371.2649	370.2571	$C_{18}H_{34}N_4O_4$	Hexamethyleneadipamide	Polyamide
15	7.10	327.2010	326.1932	$C_{14}H_{30}O_8$	Tetradecane-1,5-α,6-α,7-α,8-α,9-α,10-β,11-α-octaol	Alcohol
16	7.14	344.2275	343.2197	$C_{15}H_{29}N_5O_4$	Succinyl-leucyl-agmatine	Peptide
17	7.26	149.0121	148.0043	$C_4H_4O_6$	Dihydroxyfumaric acid	Carboxylic acid
18	7.30	324.2277	323.2199	$C_{17}H_{29}N_3O_3$	Stravidin (Streptavidin)	Protein
19	7.37	388.2536	387.2458	$C_{17}H_{33}N_5O_5$	Gln-Leu-Lys	Peptide
20	7.46	340.2591	339.2513	$C_{18}H_{33}N_3O_3$	1-Acetamido-N-{[1-(2-methoxyethyl)-4-piperidinyl]methyl}cyclohexanecarboxamide	Amide
21	7.58	432.2799	431.2721	$C_{19}H_{37}N_5O_6$	Istamycin C1	Aminoglycosides
22	7.76	476.3058	475.2980	$C_{21}H_{41}N_5O_7$	Netilmicin	Aminoglycosides
23	7.83	198.1275	197.1198	$C_9H_{15}N_3O_2$	Hercynine	Amino-acid
24	7.84	311.2060	310.1982	$C_{15}H_{26}N_4O_3$	(2R)-2-ethoxy-N-[3-(3-oxo-5,6,7,8-tetrahydro-[1,2,4]triazolo[4,3-a]pyridin-2-yl)propyl]butanamide	Amide
25	7.87	217.1069	216.0991	$C_{10}H_{16}O_5$	4-Oxosebacic acid	Carboxylic acid
26	7.96	520.3323	519.3244	$C_{30}H_{41}N_5O_3$	(3S,6S,7R,9aS)-6-[[(2S)-2-aminobutanoyl]amino]-N-benzhydryl-7-(ethylaminomethyl)-5-oxo-1,2,3,6,7,8,9,9a-octahydropyrrolo[1,2-a]azepine-3-carboxamide	Amide
27	8.06	355.2320	354.2242	$C_{17}H_{30}N_4O_4$	Ethyl (3R,4R,5S)-4-acetamido-5-(diaminomethylideneamino)-3-pentan-3-yloxycyclohexene-1-carboxylate	Ester

28	8.10	219.0752	218.0935	$C_{13}H_{14}O_3$	Eupatoriochromene	Chromene
29	8.14	453.3429	452.3351	$C_{24}H_{44}N_4O_4$	Biemamide A	Amino acid
30	8.50	273.0754	272.0676	$C_{15}H_{12}O_5$	Naringenin	Flavonoid
31	8.55	399.2586	398.2508	$C_{19}H_{34}N_4O_5$	Val-pro-ala-leu	Peptide
32	8.56	566.4268	565.4190	$C_{30}H_{55}N_5O_5$	Viscumamide	Peptide
33	8.71	443.2845	442.2767	$C_{28}H_{34}N_4O$	(3R)-N-(2,4,6-trimethylphenyl)-1-[4-(2,4,6-trimethylphenyl)pyrimidin-2-yl]piperidine-3-carboxamide	Amide
34	8.82	163.1327	162.1249	$C_8H_{18}O_3$	Octane-1,2,8-triol	Alcohol
35	8.98	548.3636	547.3558	$C_{32}H_{45}N_5O_3$	(2S,3R)-1-[4-[(1R,5S)-9-[(1S,6R)-8-bicyclo[4.3.1]decanyl]-9-azabicyclo[3.3.1]nonan-3-yl]-3-oxoquinoxalin-2-yl]-3-(dimethylamino)azetidine-2-carboxylic acid	Carboxylic acid
36	9.01	449.0708	448.0630	$C_{20}H_{16}O_{12}$	Ellagic acid 2-rhamnoside	Tannin
37	9.03	341.1380	340.1302	$C_{20}H_{20}O_5$	Licoflavanone	Flavanone
38	9.06	592.3897	591.3819	$C_{39}H_{49}N_3O_2$	N-[2,6-bis(1-methylethyl)phenyl]-3-[[2-[2-(dimethylamino)ethyl]-1-methyl- 1H-indol-3-yl]methyl]-2-oxo-3-phenyl-Cyclohexanecarboxamide	Amide
39	9.36	198.1486	197.1408	$C_{11}H_{19}NO_2$	5-(2-Oxopropyl)hygrine	Alkaloid
40	9.44	219.0471	218.0395	$C_8H_{10}O_7$	6-O-Acetylascorbic acid	Carboxylic acid
41	9.53	273.0753	272.0675	$C_{15}H_{12}O_5$	Dihydrogenistein	Isoflavonone
42	9.63	459.3030	458.2952	$C_{23}H_{42}N_2O_7$	(2S)-2-[[(2R)-2-[(2S,3S)-2-[[(2R)-2-hydroxy-3-methylbutanoyl]-methylamino]-3-methylpentanoyl]oxy-3-methylbutanoyl]-methylamino]-3-methylbutanoic acid	Carboxylic acid
43	9.74	214.0893	213.0815	$C_{13}H_{11}NO_2$	Salicylanilide	Amide
44	9.82	333.0963	332.0886	$C_{17}H_{16}O_7$	Sulochrin	Ester
45	9.88	212.1641	211.1563	$C_{12}H_{21}NO_2$	Elaeokanine C	Alkaloid
46	9.91	552.2644	551.2566	$C_{32}H_{33}N_5O_4$	5-(2-amino-6-phenylmethoxypurin-9-yl)-3-phenylmethoxy-2-(phenylmethoxymethyl)cyclopentan-1-ol	Alcohol
47	9.94	187.1327	186.1249	$C_{10}H_{18}O_3$	3-Oxodecanoic acid	Carboxylic acid
48	9.97	144.1381	143.1304	$C_8H_{17}NO$	n-Octanamide	Amide
49	10.12	149.0231	148.0153	$C_8H_4O_3$	Coumarandione	Coumarin
50	10.13	177.0544	176.0466	$C_{10}H_8O_3$	Herniarin	Coumarin
51	10.17	638.3010	637.2932	$C_{41}H_{39}N_3O_4$	1-[4-[2-(9-Butylcarbazole-3-carbonyl)-3,5-dihydroxy-3,5-dipyridin-2-ylcyclohexyl]phenyl]ethanone	Ketone
52	10.25	158.1538	157.1459	$C_9H_{19}NO$	Nonanamide	Amide
53	10.29	279.0929	278.0851	$C_{13}H_{14}N_2O_5$	Pukeleimide A	Dicarboximide
54	10.31	226.1797	225.1719	$C_{13}H_{23}NO_2$	Dichotomocej A	Diterpene
55	10.41	228.1954	227.1876	$C_{13}H_{25}NO_2$	4-Decyloxazolidin-2-one	Ketone

56	10.43	724.3370	723.3295	$C_{45}H_{45}N_3O_6$	2,2′,2″-[(2,4,6-Tributylbenzene-1,3,5-triyl)tris(methylene)]tris(1H-isoindole-1,3(2H)-dione)	Ketone
57	10.53	305.1590	304.1512	$C_{14}H_{24}O_7$	(6R)-6-[(1R)-2-Acetoxy-1-hydroxyethyl]-1-O-acetyl-3,6-anhydro-4,5-dideoxy-6-methyl-2-C-methyl-D-erythro-hexitol	Carbohydrate
58	10.55	185.0806	184.0728	$C_9H_{12}O_4$	Asperlactone	Butanolide
59	10.57	259.1536	258.1458	$C_{13}H_{22}O_5$	6-Oxoundecanedioic acid dimethyl ester	Ester
60	10.65	209.1532	208.1454	$C_{13}H_{20}O_2$	4-Heptylresorcinol	Phenol
61	10.77	200.2005	199.1928	$C_{12}H_{25}NO$	Lauramide	Amide
62	10.80	295.1900	294.1822	$C_{17}H_{26}O_4$	Embelin	Phenol
63	10.84	161.0958	160.0881	$C_{11}H_{12}O$	1-Benzosuberone	Ketone
64	11.02	172.1692	171.1615	$C_{10}H_{21}NO$	Decanamide	Amide
65	11.20	309.2056	308.1978	$C_{18}H_{28}O_4$	Ostopanic Acid	Fatty acid
66	11.37	235.1688	234.1610	$C_{15}H_{22}O_2$	Valerenic acid	Sesquiterpene
67	11.44	453.3355	452.3276	$C_{30}H_{44}O_3$	Lucialdehyde B	Triterpene
68	11.66	361.2215	360.2135	$C_{18}H_{32}O_7$	(1S,2S,3S,4S,5S,6S,7R,8R,9R,10R,12R)-6,10-Bis(hydroxymethyl)-2,3,4,8,10-pentamethyl-11-oxatricyclo[7.2.1.0~1,6~]dodecane-2,5,7,12-tetrol	Alcohol
69	11.83	310.2372	309.2293	$C_{18}H_{31}NO_3$	Melophlin M	Pyrroline
70	11.84	181.0856	180.0778	$C_{10}H_{12}O_3$	Isopropyl salicylate	Ester
71	11.86	107.0492	106.0414	C_7H_6O	Benzaldehyde	Aldehyde
72	11.87	237.1480	236.1402	$C_{14}H_{20}O_3$	Heptyl salicylate	Ester
73	12.27	228.2317	227.2238	$C_{14}H_{29}NO$	Myristamide	Amide
74	12.54	559.5188	558.5110	$C_{36}H_{66}N_2O_2$	2,5-Dimethyl-3,6-bis(tetradecylamino)-1,4-benzoquinone	Quinone
75	12.56	263.2364	262.2286	$C_{18}H_{30}O$	Farnesylacetone	Diterpenoid
76	12.61	302.2447	301.2369	$C_{20}H_{31}NO$	Eicosapentaenamide	Amide
77	12.73	280.2630	279.2552	$C_{18}H_{33}NO$	Linoleamide	Amide
78	12.82	256.2629	255.2551	$C_{16}H_{33}NO$	Palmitamide	Amide
79	12.86	563.5501	562.5420	$C_{31}H_{70}N_2O_2$	NI	-
80	12.93	304.2604	303.2527	$C_{20}H_{33}NO$	Arachidonamide	Amide
81	14.11	338.3409	337.3331	$C_{22}H_{43}NO$	Erucamide	Amide
82	14.56	536.1646	535.1568	$C_{28}H_{21}N_7O_5$	NI	-

RT = retention time; $[M+H]^+$ = measured mass as positive ion (m/z); MW = molecular mass; NI: Unidentified.

Table S5. Identification of phytochemicals compounds found in the acetonic extract of *Cochlospermum angolense* roots by HPLC-ESI-MSn.

Nº	RT	$[M+H]^+$	MW Calc	Formula	Compounds	Class of compounds
1	0.03	282.2787	281.2709	$C_{18}H_{35}NO$	(9Z)-9-Octadecenamide	Amide
2	1.12	198.0970	197.0892	$C_6H_{15}NO_6$	Aminoglucitol	aminoglycoside
3	1.14	203.0524	202.0446	$C_8H_{10}O_6$	3,5-Dioxooctanedioic acid	Carboxylic acid
4	1.15	180.0865	179.0786	$C_6H_{13}NO_5$	D-Glucosamine	Amine
5	1.16	102.1280	101.1201	$C_6H_{15}N$	Hexylamine	Amine
6	1.22	277.0890	276.0813	$C_{11}H_{16}O_8$	Siphonoside	Glycoside
7	1.23	314.1077	313.0999	$C_{11}H_{15}N_5O_6$	8-(Hydroxymethyl)guanosine	Nucleoside
8	1.26	142.1225	141.1148	$C_8H_{15}NO$	Hygrine	Alkaloid
9	1.30	191.0548	190.0470	$C_7H_{10}O_6$	3-Dehydroquinic Acid	Hydroxy acid
10	1.31	265.0914	264.0836	$C_{10}H_{16}O_8$	Kinsenoside	Glycoside
11	2.87	175.0600	174.0522	$C_7H_{10}O_5$	(+)-Shikimic acid	Carboxylic acid
12	3.77	249.0966	248.0888	$C_{10}H_{16}O_7$	3,6-Di-O-acetyl-2-deoxy-D-arabino-hexose	Carbohydrate
13	5.18	125.0389	124.0311	$C_5H_4N_2O_2$	Pyrazinoic acid	Carboxylic acid
14	5.85	219.0860	218.0782	$C_9H_{14}O_6$	Triacetin	Triglyceride
15	6.01	563.5505	562.5427	$C_{37}H_{70}N_2O_2$	N,N'-Methylenebis-(oleamide)	Amide
16	6.04	195.1225	194.1147	$C_8H_{18}O_5$	1,5-Dideoxy-2-C-[(2S)-2,3-dihydroxypropyl]-L-arabinitol	Carbohydrate
17	6.22	239.1486	238.1408	$C_{10}H_{22}O_6$	2,3,4,6-Tetra-O-methyl-D-galactitol	Carbohydrate
18	6.25	227.1751	226.1673	$C_{12}H_{22}N_2O_2$	Crotetamide	Amide
19	6.75	300.2013	299.1936	$C_{13}H_{25}N_5O_3$	L-Prolyl-L-lysylglycinamide	Peptide
20	7.03	203.0912	202.0834	$C_9H_{14}O_5$	5-Oxononanedioic acid	Carboxylic acid
21	7.11	327.2009	326.1931	$C_{14}H_{30}O_8$	Heptaethylene Glycol	Alcohol
22	7.12	344.2275	343.2197	$C_{15}H_{29}N_5O_4$	Succinyl-leucyl-agmatine	Peptide
23	7.30	324.2277	323.2199	$C_{17}H_{29}N_3O_3$	N-decanoyl L-histidine methyl ester	Ester
24	7.36	388.2535	387.2457	$C_{17}H_{33}N_5O_5$	Gln-Leu-Lys	Peptide
25	7.38	371.2272	370.2194	$C_{16}H_{34}O_9$	HO-dPEG8-OH	Alcohol
26	7.42	303.1434	302.1356	$C_{14}H_{22}O_7$	Roridinic acid	Amine
27	7.48	340.2590	339.2512	$C_{18}H_{33}N_3O_3$	1-Acetamido-N-{[1-(2-methoxyethyl)-4-piperidinyl]methyl}cyclohexanecarboxamide	Amide
28	7.58	432.2798	431.2720	$C_{19}H_{37}N_5O_6$	Istamycin C1	Aminoglycosides
29	7.73	265.1432	264.1354	$C_{15}H_{20}O_4$	(+)-Abscisic acid	sesquiterpenoid
30	7.79	476.3061	475.2983	$C_{21}H_{41}N_5O_7$	Netilmicin	aminoglicosídeo
31	7.81	311.2060	310.1982	$C_{15}H_{26}N_4O_3$	(2R)-2-ethoxy-N-[3-(3-oxo-5,6,7,8-tetrahydro-[1,2,4]triazolo[4,3-a]pyridin-2-yl)propyl]butanamide	Amide

32	7.83	198.1275	197.1197	$C_9H_{15}N_3O_2$	Hercynine	Amino-acid
33	7.87	217.1069	216.0991	$C_{10}H_{16}O_5$	4-Oxosebacic acid	Carboxylic acid
34	7.95	197.1171	196.1093	$C_{11}H_{16}O_3$	Loliolide	Quinone
35	7.96	520.3323	519.3245	$C_{24}H_{41}N_9O_4$	Benzylcarbonyl-Lys-Dab-Arg-NH2	Peptide
36	8.05	223.1325	222.1247	$C_{13}H_{18}O_3$	Hexyl salicylate	Ester
37	8.06	205.1220	204.1142	$C_{13}H_{16}O_2$	Butyl cinnamate	Ester
38	8.07	355.2320	354.2242	$C_{16}H_{34}O_8$	(4S,5S,8S,9S)-1,1,12,12-Tetramethoxy-4,5,8,9-dodecanetetrol	Alcohol
39	8.13	453.3430	452.3352	$C_{23}H_{48}O_8$	(3S,5S,7R,9R,11S,13S)-2,2,6,6,10,10,14,14-Octamethyl-1,3,5,7,9,11,13,15-pentadecaneoctol	Alcohol
40	8.25	399.2585	398.2507	$C_{18}H_{38}O_9$	Polyethyleneglycol Peg400	Alcohol
41	8.28	179.0636	178.0558	$C_5H_{10}N_2O_5$	(2S)-2-[2-(carboxymethyl)hydrazinyl]-3-hydroxypropanoic acid	Carboxylic acid
42	8.40	321.0600	320.0522	$C_{15}H_{12}O_8$	Dihydromyricetin	Flavonoid
43	8.57	566.4271	565.4193	$C_{30}H_{55}N_5O_5$	Viscumamide	peptide
44	8.58	195.1014	194.0936	$C_{11}H_{14}O_3$	Zingerone	Phenol
45	8.62	319.0809	318.0731	$C_{16}H_{14}O_7$	Dihydroisorhamnetin	Flavonoid (Flavanonol)
46	8.64	219.1377	218.1299	$C_{14}H_{18}O_2$	Pterosin B	Sesquiterpene
47	8.72	443.2845	442.2767	$C_{20}H_{42}O_{10}$	4,7,10,13,16,19,22,25-Octaoxaoctacosane-1,28-diol	Alcohol
48	8.81	163.1328	162.1250	$C_8H_{18}O_3$	1-O-Pentylglycerol	Alcohol
49	8.85	228.1494	227.1416	$C_9H_{17}N_5O_2$	4-amino-4,5-dihydro-3-methyl-5-oxo-N-pentyl-1H-1,2,4-Triazole-1-carboxamide	Amide
50	8.95	548.3635	547.3557	$C_{32}H_{45}N_5O_3$	(3S,6S,7R,9aS)-6-{[(2S)-2-Aminobutanoyl]amino}-7-[(diethylamino)methyl]-N-(diphenylmethyl)-5-oxooctahydro-1H-pyrrolo[1,2-a]azepine-3-carboxamide	Amide
51	9.00	449.0709	448.0631	$C_{20}H_{16}O_{12}$	Eschweilenol C	Phenol
52	9.05	341.1379	340.1302	$C_{20}H_{20}O_5$	(−)-8-Prenylnaringenin	Flavonoid
53	9.06	592.3898	591.3820	$C_{34}H_{49}N_5O_4$	4-[3-(1-butyltriazol-4-yl)propoxy]-N-[4-[[(2R)-5-hydroxy-1,2,3,4-tetrahydronaphthalen-2-yl]-propylamino]butyl]-3-methoxybenzamide	Amide
54	9.10	217.1432	216.1354	$C_{11}H_{20}O_4$	Undecanedioic Acid	Carboxylic acid
55	9.14	636.4159	635.4081	$C_{29}H_{57}N_5O_{10}$	[4-Amino-6-[4,6-diamino-3-[3-amino-6-(aminomethyl)-5-hydroxyoxan-2-yl]oxy-2-hydroxycyclohexyl]oxy-3,5-dihydroxyoxan-2-yl]methyl undecanoate	Ester
56	9.16	317.0288	316.0209	$C_{15}H_8O_8$	3-O-Methylellagic acid	Phenol
57	9.18	463.0866	462.0788	$C_{21}H_{18}O_{12}$	Kaempferol-3-Glucuronide	Flavonoid
58	9.22	332.2114	331.2036	$C_{17}H_{25}N_5O_2$	6-Oxoundecanedioic acid dimethyl ester	Ester
59	9.30	341.1380	340.1302	$C_{20}H_{20}O_5$	Morachalcone A	Flavonoid (Chalcone)
60	9.37	198.1486	197.1408	$C_{11}H_{19}NO_2$	N-(2-hydroxypentyl) cyclopent-3-ene-1-carboxamide	Amide
61	9.44	219.0472	218.0394	$C_8H_{10}O_7$	6-O-Acetylascorbic acid	Carboxylic acid
62	9.68	200.2008	199.1930	$C_{12}H_{25}NO$	Lauramide	Amide

63	9.70	293.2107	292.2030	$C_{18}H_{28}O_3$	Alpha-Licanic acid	Fatty acid
64	9.74	214.0893	213.0815	$C_{13}H_{11}NO_2$	Salicylanilide	Amide
65	9.82	333.0964	332.0886	$C_{17}H_{16}O_7$	Angustin B	Flavonoid
66	9.88	212.1642	211.1565	$C_{12}H_{21}NO_2$	Elaeokanine C	Alkaloid
67	9.91	552.2645	551.2565	$C_{25}H_{37}N_5O_9$	Z(OMe)-Gln-Asn-Leu-Ome	Peptide
68	9.93	187.1327	186.1249	$C_{10}H_{18}O_3$	3-Oxodecanoic acid	Carboxylic acid
69	9.96	144.1381	143.1303	$C_8H_{17}NO$	n-Octanamide	amide
70	10.09	149.0232	148.0154	$C_8H_4O_3$	Coumarandione	Coumarin
71	10.10	177.0544	176.0466	$C_{10}H_8O_3$	7-Methoxycoumarin	Coumarin
72	10.17	638.3008	637.2932	$C_{41}H_{39}N_3O_4$	2-Biphenyl-4-yl-N-(2-ethoxy-ethyl)-N-{(R)-[3-(4-ethoxy-phenyl)-4-oxo-3,4-dihydro-quinazolin-2-yl]-phenyl-methyl}-acetamide	Amide
73	10.26	158.1537	157.1459	$C_9H_{19}NO$	Nonanamide	Amide
74	10.29	226.1798	225.1720	$C_{13}H_{23}NO_2$	Dichotomocej A	Diterpene
75	10.30	279.0929	278.0851	$C_8H_{14}N_4O_7$	Diazolidinyl urea	Fatty amide
76	10.41	228.1955	227.1877	$C_{13}H_{25}NO_2$	4-Decyloxazolidin-2-one	Ketone
77	10.52	305.1590	304.1512	$C_{14}H_{24}O_7$	(6R)-6-[(1R)-2-Acetoxy-1-hydroxyethyl]-1-O-acetyl-3,6-anhydro-4,5-dideoxy-6-methyl-2-C-methyl-D-erythro-hexitol	Carbohydrate
78	10.53	185.0807	184.0729	$C_9H_{12}O_4$	Antiarol	Phenol
79	10.55	259.1536	258.1458	$C_{13}H_{22}O_5$	7-Oxo-tridecanedioic acid	Carboxylic acid
80	10.65	209.1534	208.1455	$C_{13}H_{20}O_2$	4-Heptylresorcinol	Phenol
81	10.79	295.1900	294.1822	$C_{17}H_{26}O_4$	Embelin	Phenol
82	10.81	161.0959	160.0881	$C_{11}H_{12}O$	1-Benzosuberone	Ketone
83	10.82	221.1170	220.1092	$C_{13}H_{16}O_3$	(3Z)-Hex-3-en-1-yl salicylate	Ester
84	10.84	141.0909	140.0831	$C_8H_{12}O_2$	Oct-2-ynoic acid	Carboxylic acid
85	10.87	271.1900	270.1822	$C_{15}H_{26}O_4$	Lucinone	Ketone
86	10.99	172.1694	171.1616	$C_{10}H_{21}NO$	Decanamide	Amide
87	11.19	309.2054	308.1976	$C_{18}H_{28}O_4$	Ostopanic Acid	Fatty acid
88	11.34	291.1948	290.1870	$C_{18}H_{26}O_3$	Octyl methoxycinnamate	Ester
89	11.37	235.1688	234,1610	$C_{15}H_{22}O_2$	Valerenic acid	Sesquiterpene
90	11.44	453.3355	452.3277	$C_{30}H_{44}O_3$	Ganoderic acid S	Triterpenoid
91	11.66	259.1536	258.1458	$C_{13}H_{22}O_5$	6-Oxo-undecanedioic acid dimethyl ester	Ester
92	11.67	361.2214	360.2136	$C_{18}H_{32}O_7$	Methyl 2,4-di-O-acetyl-3-O-octylpentopyranoside	Carbohydrate
93	11.76	200.2003	199.1925	$C_{12}H_{25}NO$	N-Decylacetamide	Amide
94	11.84	310.2372	309.2294	$C_{18}H_{31}NO_3$	Melophlin M	Pyrroline

95	11.86	107.0492	106.0415	C_7H_6O	Benzaldehyde	Aldehyde
96	12.32	228.2316	227.2238	$C_{14}H_{29}NO$	Myristamide	Amide
97	12.54	263.2365	262.2287	$C_{18}H_{30}O$	Farnesylacetone	Diterpenoid
98	12.57	280.2628	279.2550	$C_{18}H_{33}NO$	Linoleamide	Amide
99	12.61	302.2448	301.2370	$C_{20}H_{31}NO$	Deramciclane	Anxiolytic
100	12.81	256.2628	255.2550	$C_{16}H_{33}NO$	Palmitamide	Amide
101	12.85	563.5502	562.5424	$C_{36}H_{70}N_2O_2$	1,1'-[(E)-Diazenediyl]di(octadecan-1-one)	Ketone
102	12.92	304.2604	303.2526	$C_{20}H_{33}NO$	Arachidonamide	Amide
103	14.20	338.3410	337.3332	$C_{22}H_{43}NO$	Erucamide	Amide

RT = retention time; $[M+H]^+$ = measured mass as positive ion (m/z); MW = molecular mass.

Table S6. Identification of phytochemicals compounds found in the ethanolic extract of *Cochlospermum angolense* roots by HPLC-ESI-MSn.

Nº	RT	$[M+H]^+$	MW Calc	Formula	Compounds	Class of compounds
1	0.03	282.2788	281.2710	$C_{18}H_{35}NO$	(9Z)-9-Octadecenamide	Amide
2	1.05	162.0760	161.0682	$C_6H_{11}NO_4$	2-Aminohexanedioic acid	Amino acid
3	1.06	342.1388	341.1310	$C_{12}H_{23}NO_{10}$	Lactosylamine	Amine
4	1.10	198.0970	197.0892	$C_6H_{15}NO_6$	Aminoglucitol	Amino glycoside
5	1.14	180.0864	179.0786	$C_6H_{13}NO_5$	D-Glucosamine	Amine
6	1.16	203.0523	202.0445	$C_8H_{10}O_6$	3,5-Dioxooctanedioic acid	Carboxylic acid
7	1.21	365.1049	364.0971	$C_{14}H_{20}O_{11}$	(3R,4R,5R)-5-[(2R,3R,4S,5S,6R)-6-(formyloxymethyl)-3,4,5-trihydroxyoxan-2-yl]oxy-3,4-dihydroxycyclohexene-1-carboxylic acid	Carboxylic acid
8	5.03	125.0390	124.0312	$C_5H_4N_2O_2$	Pyrazinoic acid	Carboxylic acid
9	5.58	195.1226	194.1148	$C_8H_{18}O_5$	(2R,3R,4R,5R)-6-Methyl-1,2,3,4,5-heptanepentol	Alcohol
10	5.92	227.1752	226.1674	$C_{12}H_{22}N_2O_2$	Crotetamide	Amide
11	5.95	217.1044	216.0966	$C_{10}H_{16}O_5$	4-Oxosebacic acid	Carboxylic acid
12	6.22	239.1486	238.1408	$C_{10}H_{22}O_6$	2,3,4,6-Tetra-O-methyl-D-galactitol	Carbohydrate
13	6.38	563.5505	562.5427	$C_{36}H_{70}N_2O_2$	1,1'-[(E)-Diazenediyl]di(octadecan-1-one)	Ketone
14	6.75	300.2013	299.1935	$C_{13}H_{25}N_5O_3$	L-Prolyl-L-lysylglycinamide	Peptide
15	7.10	327.2009	326.1931	$C_{14}H_{30}O_8$	Heptaethylene Glycol	Alcohol
16	7.12	344.2275	343.2196	$C_{15}H_{29}N_5O_4$	Succinyl-leucyl-agmatine	Peptide
17	7.30	324.2278	323.2200	$C_{17}H_{29}N_3O_3$	Stravidin	Peptide
18	7.36	371.2272	370.2194	$C_{16}H_{34}O_9$	3,6,9,12,15,18,21-Heptaoxatricosane-1,23-diol	Alcohol
19	7.38	388.2537	387.2457	$C_{17}H_{33}N_5O_5$	Gln-Leu-Lys	Peptide
20	7.46	340.2591	339.2514	$C_{18}H_{33}N_3O_3$	1-Acetamido-N-{[1-(2-methoxyethyl)-4-piperidinyl]methyl}cyclohexanecarboxamide	Amide
21	7.57	432.2797	431.2719	$C_{26}H_{33}N_5O$	N5-((2-((3-isopropyl-3-azabicyclo[3.2.1]octan-8-yl)methoxy)pyridin-4- yl)methyl)isoquinoline-1,5-diamine	Amine
22	7.79	476.3060	475.2982	$C_{21}H_{41}N_5O_7$	Netilmicin	Amino glycoside
23	7.82	311.2061	310.1983	$C_{14}H_{30}O_7$	3,6,9,12,15,18-Hexaoxaicosan-1-ol	Alcohol
24	7.84	198.1276	197.1198	$C_9H_{15}N_3O_2$	Hercynine	Amino-acid
25	7.87	217.1068	216.0990	$C_{10}H_{16}O_5$	1,4-Diethyl 2-acetylbutanedioate	Ester
26	7.95	520.3323	519.3245	$C_{23}H_{45}N_5O_8$	2-[4-amino-3-[3-amino-6-[(2,3-dihydroxypropylamino)methyl]-3,4-dihydro-2H-pyran-2-yl]-2-hydroxy-6-(methylamino)cyclohexyl]oxy-5-methyl-4-(methylamino)oxane-3,5-diol	Amide
27	8.12	170.1175	169.1097	$C_9H_{15}NO_2$	N-Pentylsuccinimide	Amide
28	8.13	453.3431	452.3353	$C_{23}H_{48}O_8$	(3S,5S,7R,9R,11S,13S)-2,2,6,6,10,10,14,14-Octamethyl-1,3,5,7,9,11,13,15-pentadecaneoctol	Alcohol

29	8.16	311.2059	310.1981	$C_{14}H_{30}O_7$	3,6,9,12,15,18-Hexaoxaicosan-1-ol	Alcohol
30	8.35	355.2322	354.2244	$C_{16}H_{34}O_8$	4,7,10,13,16,19-Hexaoxadocosane-1,22-diol	Alcohol
31	8.37	179.0636	178.0558	$C_5H_{10}N_2O_5$	(2S)-2-[2-(carboxymethyl)hydrazinyl]-3-hydroxypropanoic acid	Carboxylic acid
32	8.52	399.2586	398.2508	$C_{18}H_{38}O_9$	Polyethylene glycol-9-ethoxylate	Ester
33	8.55	566.4269	565.4192	$C_{30}H_{55}N_5O_5$	Viscumamide	Amide
34	8.63	219.1378	218.1300	$C_{14}H_{18}O_2$	Pterosin B	Sesquiterpenoid
35	8.71	443.2848	442.2769	$C_{20}H_{42}O_{10}$	2,5,8,11,14,17,20,23,26,29-Decaoxatriacontane	Ether
36	8.74	241.2034	240.1956	$C_{12}H_{24}N_4O$	2-{3-[2-(Diethylamino)ethyl]-1,2,4-oxadiazol-5-yl}-N-methyl-2-propanamine	Amine
37	8.80	163.1328	162.1250	$C_8H_{18}O_3$	Diethylene glycol n-butyl ether	Ether
38	8.83	228.1494	227.1416	$C_9H_{17}N_5O_2$	2-[(6-Hydrazinyl-5-methylpyridazin-3-yl)-(2-hydroxyethyl)amino]ethanol	Alcohol
39	8.86	504.3370	503.3292	$C_{30}H_{41}N_5O_2$	1-(4-(3-((3aR,6aS)-5-(4,6-dimethylpyrimidine-5-carbonyl)hexahydropyrrolo[3,4-c]pyrrol-2(1H)-yl)-1-phenylpropyl)piperidin-1-yl)propan-1-one	Ketone
40	8.90	340.2590	339.2512	$C_{23}H_{33}NO$	Evocarpine	Akaloid
41	8.96	185.1145	184.1076	$C_{10}H_{16}O_3$	9-Oxodecenoic acid	Carboxylic acid
42	8.97	548.3635	547.3557	$C_{32}H_{45}N_5O_3$	(3S,6S,7R,9aS)-6-[[(2S)-2-aminobutanoyl]amino]-N-benzhydryl-7-(diethylaminomethyl)-5-oxo-1,2,3,6,7,8,9,9a-octahydropyrrolo[1,2-a]azepine-3-carboxamide	Amide
43	9.06	592.3896	591.3821	$C_{39}H_{49}N_3O_2$	Carbamic acid, [1,1'-biphenyl]-2-yl-, 4-methyl-1-[8-[methyl(1-naphthalenylmethyl)amino]octyl]-4-piperidinyl ester	Ester
44	9.11	217.1433	216.1355	$C_{11}H_{20}O_4$	Undecanedioic Acid	Carboxylic acid
45	9.15	636.4159	635.4081	$C_{41}H_{53}N_3O_3$	4-[[4-(Diethylamino)-2-methylphenyl]imino]-N-[3-(2,4-di-tert-pentylphenoxy)propyl]-1-oxonaphthalene-2-carboxamide	Amide
46	9.22	332.2112	331.2034	$C_{17}H_{25}N_5O_2$	(R)-Prizidilol	Alcohol
47	9.23	680.4420	679.4342	$C_{43}H_{57}N_3O_4$	(4Z)-4-[(4-methoxyphenyl)hydrazinylidene]-1-oxo-N-[2-(3-pentadecylphenoxy)butyl]naphthalene-2-carboxamide	Amide
48	9.28	354.2243	353.2165	$C_{19}H_{31}NO_5$	Cinnabaramide E	Amide
49	9.31	234.0581	233.0503	$C_8H_{11}NO_7$	N-(Carboxyacetyl)-L-glutamic acid	Amino-acid
50	9.37	198.1487	197.1409	$C_{11}H_{19}NO_2$	1-(2-Ethyl-1-piperidinyl)-1,3-butanedione	Ketone
51	9.44	219.0472	218.0394	$C_8H_{10}O_7$	6-O-Acetylascorbic acid	Carboxylic acid
52	9.54	200.2008	199.1930	$C_{12}H_{25}NO$	Lauramide	Amide
53	9.73	214.0893	213.0815	$C_{13}H_{11}NO_2$	Salicylanilide	Amide
54	9.83	333.0964	332.0886	$C_{17}H_{16}O_7$	Blumeatin B	Flavonoid
55	9.87	212.1642	211.1564	$C_{12}H_{21}NO_2$	Elaeokanine C	Alkaloid
56	9.91	552.2643	551.2565	$C_{37}H_{33}N_3O_2$	N-{1-[3-(3-methylphenyl)-4-oxo-3,4-dihydroquinazolin-2-yl]propyl}-N-(2-phenylethyl)naphthalene-2-carboxamide	Amide

57	9.93	187.1326	186.1248	$C_{10}H_{18}O_3$	3-Oxodecanoic acid	Carboxylic acid
58	9.95	144.1382	143.1304	$C_8H_{17}NO$	n-Octanamide	Amide
59	10.09	149.0232	148.0154	$C_8H_4O_3$	Coumarandione	Coumarin
60	10.10	177.0544	176.0466	$C_{10}H_8O_3$	7-Methoxycoumarin	Coumarin
61	10.17	638.3011	637.2933	$C_{41}H_{39}N_3O_4$	1-[4-[2-(9-Butylcarbazole-3-carbonyl)-3,5-dihydroxy-3,5-dipyridin-2-ylcyclohexyl]phenyl]ethanone	Ketone
62	10.25	158.1538	157.1459	$C_9H_{19}NO$	Nonanamide	Amide
63	10.28	279.0929	278.0851	$C_{13}H_{14}N_2O_5$	Pukeleimide A	Amide
64	10.30	226.1798	225.1720	$C_{13}H_{23}NO_2$	Dichotomocej A	Diterpenoid
65	10.42	228.1954	227.1876	$C_{13}H_{25}NO_2$	4-Decyloxazolidin-2-one	Ketone
66	10.44	724.3375	723.3297	$C_{45}H_{45}N_3O_6$	2,2′,2″-[(2,4,6-Tributylbenzene-1,3,5-triyl)tris(methylene)]tris(1H-isoindole-1,3(2H)-dione)	Ketone
67	10.52	305.1590	304.1512	$C_{14}H_{24}O_7$	(6R)-6-[(1R)-2-Acetoxy-1-hydroxyethyl]-1-O-acetyl-3,6-anhydro-4,5-dideoxy-6-methyl-2-C-methyl-D-erythro-hexitol	Carbohydrate
68	10.53	259.1537	258.1459	$C_{13}H_{22}O_5$	6-Oxo-undecanedioic acid dimethyl ester	Ester
69	10.54	185.0807	184.0729	$C_9H_{12}O_4$	Umbellatolide B	Terpene (Terpenoid)
70	10.64	209.1533	208.1455	$C_{13}H_{20}O_2$	(-)-Theaspirone	Ketone
71	10.80	161.0959	160.0881	$C_{11}H_{12}O$	1-Benzosuberone	Ketone
72	10.82	295.1899	294.1821	$C_{17}H_{26}O_4$	Embelin	Phenol
73	10.99	172.1694	171.1616	$C_{10}H_{21}NO$	Decanamide	Amide
74	11.00	239.1614	238.1536	$C_{14}H_{22}O_3$	7-Oxo-11E,13-Tetradecadienoic acid	Carboxylic acid
75	11.18	309.2055	308.1977	$C_{18}H_{28}O_4$	5-O-Methyl embelin	Phenol
76	11.32	291.1949	290.1871	$C_{18}H_{26}O_3$	Octyl methoxycinnamate	Ester
77	11.35	235.1690	234.1612	$C_{15}H_{22}O_2$	Valerenic acid	Sesquiterpenoid
78	11.44	453.3354	452.3276	$C_{30}H_{44}O_3$	Lucialdehyde B	Terpene (Triterpenoid)
79	11.66	361.2213	360.2135	$C_{18}H_{32}O_7$	Methyl 2,4-di-O-acetyl-3-O-octylpentopyranoside	Carbohydrate
80	11.69	259.1535	258.1457	$C_{13}H_{22}O_5$	7-Oxo-tridecanedioic acid	Carboxylic acid
81	11.80	310.2371	309.2292	$C_{18}H_{31}NO_3$	Melophlin M	Ketone
82	11.84	107.0493	106.0415	C_7H_6O	Benzaldehyde	Aldehyde
83	11.86	237.1481	236.1403	$C_{14}H_{20}O_3$	Heptyl salicylate	Ester
84	12.19	228.2319	227.2241	$C_{14}H_{29}NO$	Myristamide	Amide
85	12.25	304.2606	303.2528	$C_{20}H_{33}NO$	Arachidonamide	Amide
86	12.54	263.2363	262.2285	$C_{18}H_{30}O$	Farnesylacetone	Terpeno (Sesquiterpeno)
87	12.56	280.2628	279.2550	$C_{18}H_{33}NO$	Linoleamide	Amide
88	12.58	559.5189	558.5111	$C_{36}H_{66}N_2O_2$	2,5-Dimethyl-3,6-bis(tetradecylamino)-1,4-benzoquinone	Quinone

89	12.80	256.2629	255.2551	$C_{16}H_{33}NO$	Palmitamide	Amide
90	14.08	338.3411	337.3333	$C_{22}H_{43}NO$	Erucamide	Amide

RT = retention time; $[M+H]^+$ = measured mass as positive ion (m/z); MW = molecular mass.

Table S7. Identification of common phytochemicals found in acetonic and ethanolic extracts of leaves, barks and roots of *Cochlospermum angolense* by HPLC-ESI-MSn. (+) Present; (-) Absent.

Nº	TR	$[M+H]^+$	Molecular formula	Compounds	Organic class	Leaves	Barks	Roots
1	7.73	265.1432	$C_{15}H_{20}O_4$	(+)-Abscisic acid	Sesquiterpenoid	-	-	+
2	7.95	197.1171	$C_{11}H_{16}O_3$	Loliolide	Quinone	-	+	+
3	7.97	449.1073	$C_{21}H_{20}O_{11}$	Quercetin	Flavonol	+	-	-
4	7.97	449.1073	$C_{21}H_{20}O_{11}$	Quercitrin	Flavonoid glycoside	+	-	-
5	7.98	306.2460	$C_{19}H_{31}NO_2$	Samandarin	Alkaloid	+	-	-
6	8.10	449.1072	$C_{21}H_{20}O_{11}$	Astragalin	Flavonoid glycoside	+	-	-
7	8.11	219.1013	$C_{13}H_{14}O_3$	Eupatoriochromene	Chromene	-	+	-
8	8.20	433.1125	$C_{21}H_{20}O_{10}$	Vitexin	Flavone glycoside	+	-	-
9	8.38	433.1122	$C_{21}H_{20}O_{10}$	Apigetrin	Flavonoid glycoside	+	-	-
10	8.40	321.0600	$C_{15}H_{12}O_8$	Dihydromyricetin	Flavanonol	-	+	+
11	8.47	183.1014	$C_{10}H_{14}O_3$	Hydroconiferyl Alcohol	Flavonoid	+	-	-
12	8.47	273.0752	$C_{15}H_{12}O_5$	Naringenin	Flavonone		+	-
13	8.55	479.0813	$C_{21}H_{18}O_{13}$	Quercetin-3'-glucuronide	Flavonoid glycoside	+	-	-
14	8.58	195.1014	$C_{11}H_{14}O_3$	Zingerone	Phenol	-	-	+
15	8.60	465.1023	$C_{21}H_{20}O_{12}$	Isoquercetin	Flavonoid glycoside	+	-	-
16	8.62	289.0703	$C_{15}H_{12}O_6$	(+)-Dihydrokaempferol	Flavonoid	-	+	-
17	8.62	319.0809	$C_{16}H_{14}O_7$	Dihydroisorhamnetin	Flavanone	-	+	+
18	8.63	219.1378	$C_{14}H_{18}O_2$	Pterosin B	Sesquiterpenoid	-	-	+
19	8.70	303.0495	$C_{15}H_{10}O_7$	Morin	Flavonol	+	-	-
20	8.73	435.0916	$C_{20}H_{18}O_{11}$	Quercetin-3-Arabinoside	Flavonoid glycoside	+	-	-
21	8.90	340.2590	$C_{23}H_{33}NO$	Evocarpine	Alkaloid	-	-	+
22	8.95	287.0545	$C_{15}H_{10}O_6$	Kaempferol	Flavonol	+	-	-
23	8.98	133.0648	C_9H_8O	Cinnamaldehyde	Aldehyde	+	-	-
24	8.98	371.1484	$C_{21}H_{22}O_6$	(+)-Fargesin	Lignan	-	+	-
25	9.00	449.0709	$C_{20}H_{16}O_{12}$	Eschweilenol C	Phenol	-	-	+
26	9.01	449.0708	$C_{20}H_{16}O_{12}$	Ellagic acid 2-rhamnoside	Tannin	-	+	-
27	9.03	341.1377	$C_{20}H_{20}O_5$	(−)-8-Prenylnaringenin	Flavanone	-	+	+
28	9.03	341.1380	$C_{20}H_{20}O_5$	Dihydrogenistein	Isoflavonone	-	+	-
29	9.08	592.3895	$C_{33}H_{53}NO_8$	Edpetiline	Alkaloid	-	+	-

30	9.13	363.1644	$C_{16}H_{26}O_9$	Oleuropeinol	Phenol acid	+	-	-
31	9.16	317.0288	$C_{15}H_8O_8$	3-O-Methylellagic acid	Phenol	-	-	+
32	9.18	463.0866	$C_{21}H_{18}O_{12}$	Kaempferol-3-Glucuronide	Flavonoid	-	-	+
33	9.31	341.1378	$C_{20}H_{20}O_5$	Morachalcone A	Chalcone	-	+	+
34	9.36	198.1486	$C_{11}H_{19}NO_2$	5-(2-Oxopropyl) hygrine	Alkaloid	-	+	
35	9.52	273.0753	$C_{15}H_{12}O_5$	Pinobanksin	Flavanol	-	+	+
36	9.82	333.0964	$C_{17}H_{16}O_7$	Angustin B	Flavonoid	-	-	+
37	9.83	333.0964	$C_{17}H_{16}O_7$	Blumeatin B	Flavonoid	-	-	+
38	9.83	333.0964	$C_{17}H_{16}O_7$	Licoflavanone	Flavonone	-	+	
39	9.90	212.1641	$C_{12}H_{21}NO_2$	Elaeokanine C	Alkaloid	-	+	+
40	10.09	177.0544	$C_9H_{12}O_4$	7-Methoxycoumarin (Herniarin)	Coumarin	+	+	+
41	10.12	149.0230	$C_8H_4O_3$	Coumaranone	Coumarin	+	+	+
42	10.21	200.1643	$C_{11}H_{21}NO_2$	5-(2-Hydroxypropyl)-hygrine	Alkaloid	-	+	-
43	10.34	226.1796	$C_{13}H_{23}NO_2$	Dichotomocej A	Diterpene	+	+	-
44	10.53	185.0805	$C_9H_{12}O_4$	Antiarol	Phenol	+	-	-
45	10.54	185.0807	$C_9H_{12}O_4$	Umbellatolide B	Terpenoid	-	-	+
46	10.55	305.1588	$C_{14}H_{24}O_7$	Urechitol B	Terpenoid	+	-	-
47	10.63	209.1533	$C_{13}H_{20}O_2$	4-Heptylresorcinol	Phenol	+	+	+
48	10.65	209.1533	$C_{13}H_{20}O_2$	5-Heptylresorcinol	Phenol	+	-	-
49	10.79	295.1900	$C_{17}H_{26}O_4$	Embelin	Phenol	+	+	+
50	10.80	295.1899	$C_{17}H_{26}O_4$	(+)-[6]-gingerol	Phenol	-	+	-
51	11.09	237.1845	$C_{15}H_{24}O_2$	Farnesoic acid	Terpenoid	+	-	-
52	11.21	309.2054	$C_{18}H_{28}O_4$	5-O-Methyl embelin (monohydroxi-1,4-benzoquinone)	Quinone	-	+	-
53	11.35	235.1690	$C_{15}H_{22}O_2$	Drimenin	Terpene	-	+	-
54	11.35	235.1689	$C_{15}H_{22}O_2$	Valerenic acid	Sesquiterpeno	+	+	+
55	11.42	453.3355	$C_{30}H_{44}O_3$	Kulactone	Triterpenoid	-	+	-
56	11.44	453.3355	$C_{30}H_{44}O_3$	Ganoderic acid S	Triterpenoid	-	+	-
57	11.44	453.3355	$C_{30}H_{44}O_3$	Lucialdehyde B	Triterpene	-	+	-
58	11.84	107.0493	C_7H_6O	Benzaldehyde	Aldehyde	+	+	+
59	12.55	263.2364	$C_{18}H_{30}O$	Farnesylacetone	Sesquiterpene Ketone	-	+	+
60	12.59	559.5189	$C_{36}H_{66}N_2O_2$	2,5-Dimethyl-3,6-bis(tetradecylamino)-1,4-benzoquinone	Quinone	-	+	+

RT = retention time; $[M+H]^+$ = measured mass as positive ion (m/z); MW = molecular mass; E.A = acetone extract; E.E = ethanol extract.
